# Predicting affinity and potency of new psychoactive substances at cannabinoid 1 receptor with explainable artificial intelligence

**DOI:** 10.3389/fphar.2026.1815814

**Published:** 2026-05-20

**Authors:** Verena Schöning, Gaia Alluisetti, Katharina Elisabeth Grafinger, Daniel Pasin, Christophe P. Stove, Henrik Gréen, Wolfgang Weinmann, Felix Hammann

**Affiliations:** 1 Clinical Pharmacology and Toxicology, Department of Internal Medicine, University Hospital Bern, Bern, Switzerland; 2 Institute of Forensic Medicine Bern, Forensic Toxicology and Chemistry, University of Bern, Bern, Switzerland; 3 Graduate School for Cellular and Biomedical Sciences, University of Bern, Bern, Switzerland; 4 Hyperion Data, Griffith, NSW, Australia; 5 Laboratory of Toxicology, Department of Bioanalysis, Faculty of Pharmaceutical Sciences, Ghent University, Ghent, Belgium; 6 Division of Clinical Chemistry and Pharmacology, Department of Biomedical and Clinical Sciences, Linköping University, Linköping, Sweden; 7 Department of Forensic Genetics and Forensic Toxicology, National Board of Forensic Medicine, Linköping, Sweden

**Keywords:** binding affinity, CB1, explainable artificial intelligence, machine learning, NPS, potency, SHAP values

## Abstract

**Introduction:**

Binding affinity and functional potency are two distinct but pharmacologically related properties in ligand-receptor interactions. The molecular features influencing these two endpoints may differ, reflecting distinct physicochemical and conformational requirements for receptor binding and activation. Consequently, understanding the molecular features that determine both endpoints is essential, especially for the regulation of fast-moving markets of recreational drugs such as new psychoactive substances (NPS), where manufacturers are able to circumvent the stringent national and international regulations by exploiting loopholes within drug regulation. Synthetic cannabinoid receptor agonists form one class of NPS and are generally full agonists of the cannabinoid 1 receptor (CB1).

**Methods:**

We compiled publicly available data on CB1 affinity and potency. We used molecular descriptors and fingerprints to train five machine learning (ML) classification models. Using explainable Artificial Intelligence, particularly SHAP values, we analyzed the features driving affinity and potency.

**Results:**

Especially XGBoost (XGB) and Random Forest (RF) with molecular descriptors and Extended Connectivity Fingerprints (ECFP), respectively, outperformed the other models in predicting binding affinity. For potency, XGB and RF showed excellent performance with either molecular descriptors or ECFP. All these models had recall, precision, and F1 scores >90%. Further, we mapped SHAP values to selected NPS to highlight structural features having a positive or negative impact on high affinity and potency.

**Discussion:**

Affinity relies mainly on lipophilicity and membrane-partitioning descriptors, whereas potency depends on a broader combination of lipophilicity, shape, branching, and electronic descriptors.

## 1 Introduction

Binding affinity and functional potency of a molecule to a specific receptor are important metrics, as ligand-receptor interactions are crucial for the pharmacological effects, e.g., signal transmission and facilitation of biological functions. While binding affinity reflects the thermodynamic strength of ligand–receptor interaction, potency describes the downstream functional consequences of that binding, including receptor activation, signaling cascades, and system-level responses. Binding affinity is typically measured by the inhibition constant (Ki), whereas potency is described by the half maximal effective concentration (EC_50_). However, despite the pharmacological relationship between affinity and potency, the molecular features that influence these endpoints may differ, reflecting distinct physicochemical and conformational requirements for receptor binding and activation. Consequently, understanding the molecular features that determine both endpoints is essential for rational and purposeful drug design.

Numerous studies have shown that machine learning (ML) is suitable to model ligand–receptor interactions. It facilitates high-throughput prediction of affinity and potency from molecular features and structure, e.g., activation of AMP-dependent protein kinase (AMPK) by plant constituents (Drewe et al., 2021; Drewe et al., 2024). The integration of explainable Artificial Intelligence (xAI) methods not only enables accurate prediction but also provides mechanistic interpretations of structure–activity relationships, enabling the identification of key molecular substructures that modulate receptor interactions.

High-throughput prediction of binding affinity and potency is especially important in fast-moving markets of recreational drugs, such as new psychoactive substances (NPS). NPS are a heterogeneous group of small molecules that mimic the actions and psychoactive effects of existing pharmaceutical products or recreational drugs (Shafi et al., 2020; Chatwin, 2017). Due to the ongoing, rapid development of chemical analogues and newly synthesized substances, manufacturers of NPS can circumvent stringent national and international drug regulations by exploiting loopholes, leaving the legislative process behind (Santos et al., 2024). One class of NPS are synthetic cannabinoid receptor agonists (SCRAs), which are generally full agonists of the cannabinoid 1 receptor (CB_1_), a G-protein-coupled receptor (GPCR). SCRAs are associated with multiple, potentially life-threatening, adverse effects such as psychosis, anxiety, seizures, agitation, hypothermia, tachycardia, and kidney injury (Tait et al., 2016), and non-fatal and fatal intoxications are frequently reported (Groth et al., 2023; Simon et al., 2023).

Several ML studies were already conducted focusing on affinity prediction at CB_1_. One of the earliest studies used 703 substances to predict CB_1_ and CB_2_ (cannabinoid 2 receptor) selectivity, based on their respective CB_1_ and CB_2_ affinities. The selectivity for each receptor was defined as the difference in the affinities, i.e., a more than 10-fold difference was the threshold for selectivity. The compounds in the training dataset were described using four fingerprint types. Based on these data, an adaptive boosting (Ligand Classifier of Adaptively Boosting Ensemble Decision Stumps) and a support vector machine classification model were trained. All models had a modest precision and recall with values mainly between 60% and 70% (Ma et al., 2013). In a study to predict the affinity of SCRAs, 1770 compounds were compiled, and 210 CATS (chemically advanced template search) descriptors were calculated. The quantitative affinity of an unknown substance was predicted by identifying sufficiently similar neighbors and interpolating the value based on weighted distances. The predictive power of the approach was evaluated solely using the coefficient of linear correlation after log-transforming the affinity (Paulke et al., 2016). Another study trained supervised classification models to predict the orthosteric ligands and allosteric modulators for CB_1_ and CB_2_. The cut-off between active and inactive molecules was set to Ki = 100 nM. Molecular descriptors and two different types of fingerprints were used as features and 7 ML algorithms were compared. All models showed imbalanced performance, characterized by high recall but limited precision, suggesting that while they capture most positive cases, they lack specificity (Bian et al., 2019). A partial least squares regression model was trained to predict the CB_1_ affinity, expressed as pKi (negative logarithm (base of 10) of Ki), of SCRAs using molecular descriptors. The measured binding affinities in the dataset ranged from 22 to 12,000 nM. However, the dataset consisted of only 15 substances. This restricted chemical space was evident during external validation with 62 naphthoylindole cannabinoids, which adversely affected prediction performance (Lee et al., 2020). A more comprehensive training dataset with 1,958 and 2,616 substances for CB_1_ and CB_2_, respectively, was used to train regression models based on molecular descriptors and fingerprints. A gradient boosting ensemble was used to predict pKi values and create an embedded descriptor space. Using this embedded representation, a k-nearest neighbor algorithm was used to predict an average pKi, provided that all neighbors were within a defined Euclidean distance (Mizera et al., 2020). A curated dataset containing the pKi values of 3,846 for human CB_1_ and 3,514 substances for human CB_2_ was used to analyze the affinity and selectivity using atom pair fingerprints. For 2,183 substances, information for both receptors were available. For each receptor, two different classification models were trained with different pKi thresholds. To analyze the selectivity of a substance, three classes (CB_1_ selective, non-selective, CB_2_ selective) were created based on the difference in pKi values and again trained classification models. For each classification model, different ML algorithms were compared, i.e., Random Forest (RF), k-nearest neighbors (kNN), Gradient Boosting, eXtreme Gradient Boosting (XGB), and Multi-Layer Perceptron (MLP). Overall, RF had the best predictive performance with a balanced accuracy ranging from 0.7 (for the external validation) up to 0.9 (internal validation) (Delre et al., 2023). A multilayer, RF classification model was trained to distinguish selective and non-selective CB_1_ and CB_2_ ligands. The substances were described using core-substituent fingerprints (CSFP). Furthermore, xAI methodology was applied to identify the characteristic substructures in isoform-selective ligands (Gambacorta et al., 2023). To the best of our knowledge, no studies have been conducted to predict ligand potency at CB_1_ using ML. However, as SCRAs, like other NPS, can be highly potent, with their potency even exceeding that of the template prototypical drug (Sachdev et al., 2019; Waters et al., 2024), the chemical space defined by the underlying dataset for the ML models needs to be selected accordingly.

In this study, we employ explainable ML models, focusing on high-affinity and high-potency ligands for CB_1_, to predict and compare the physicochemical and structural features that drive these two distinct yet related (i.e., binding is a prerequisite for activity, but does not suffice for activity) pharmacological properties. By applying xAI techniques, i.e., SHAP values, we identify and contrast the molecular features that underlie binding strength and receptor activation.

## 2 Materials and methods

### 2.1 Data compilation

We downloaded binding affinity and potency data for the CB_1_ from PubChem (https://www.ncbi.nlm.nih.gov/pcassay/) during September 2024. We vetted entries against original literature. If brain matter was used, but the type of CB receptor was not mentioned, we assumed it to be CB_1_ (Pertwee, 1997). If we found discrepancies, i.e., unclear results (e.g., contradictory information within the publication, unclear decimal places), or wrong records (e.g., experiments related to the CB_2_), we excluded these results. We further excluded results that were not obtained from *in vitro* experiments (e.g., machine learning predictions, *in vivo,* or *ex vivo* assays) or that were cited from other studies. In addition, if research articles were mentioned in any of the literature, we used for vetting that were not part of our database, we checked them for eligibility and, if possible, included the information. Furthermore, we searched PubMed (https://pubmed.ncbi.nlm.nih.gov/) for any literature published from 2020–2025 concerning the affinity and potency of substances to CB_1_.

### 2.2 Pre-processing

We used the *molvs* package (version 0.1.1), a Python tool that leverages RDKit to validate and standardize molecular information, to standardize SMILES and mitigate issues arising from SMILES from different sources. To remove counterions and other additives, we retained only the largest fragment of each substance. We calculated different molecular descriptors, i.e., PaDEL (Yap, 2011) (length = 1,444), and fingerprint descriptors, i.e., MACCS (Molecular Access System by Molecular Design Limited, length = 166), ECFP (extended connectivity fingerprints, Morgan fingerprints with radius of 3, length = 2,048) (Rogers and Hahn, 2010), and KRFP (Klekota Roth fingerprints, length = 4,860) (Klekota and Roth, 2008).

We performed an unspecific (outcome-independent) feature selection to reduce the dimensionality of the datasets. The following steps were conducted for the molecular descriptor dataset.

- Removal all columns with more than 10 non-available (NA) values (i.e., values that were not computed);
- Removal of all rows with NAs (0.2%), resulting in a table with no NA values;
- Removal of columns with low variability, defined as less than 10% unique values;
- Removal of all but one highly correlated descriptor, with a threshold of 0.95 (the threshold was chosen based on experience).

As fingerprint descriptors are binary (feature present or absent), we dropped columns with constant values (the same value for all rows) from these datasets.

If a substance was reported multiple times (e.g., across different publications or assays), only the entry with the highest reported affinity or potency was retained. From a toxicological standpoint, this is a conservative approach, which prioritizes public health by intentionally overestimating risk, using safety factors, and assuming high toxicity when data is limited or conflicting.

We split the datasets into a training (80%) and a test (20%) dataset, while maximizing the chemical space based on molecular features. For this, we calculated Morgan fingerprints (radius of 2) of the substances and then used a specific picking strategy aimed at diversity of the dataset (Ashton et al., 2002), while also stratifying for the outcome. Thus, the resulting split maximized the diversity of the datasets with regard to molecular features. To avoid data leakage, we calculated the nearest-neighbor Tanimoto similarity using ECFP and removed substances with a value of 1.0 from the test dataset.

### 2.3 Outcome engineering

Due to the high variability within the experimental affinity and potency data, we decided to train binned (n-ary) classification models. We compared different thresholds for single classes, introduced gaps of varying sizes between classes, and evaluated approaches for handling data flagged as unspecific or inconclusive by PubChem to optimize our class definition.

### 2.4 Comparison of ML algorithms and predictor data sets

We trained and compared five different machine learning algorithms, i.e., eXtreme gradient boosting (XGB), random forest (RF), support vector machines (SVM), multi-layer perceptron (MLP), and logistic regression (LR), using the four different calculated molecular descriptors and fingerprint datasets (molecular descriptors, MACCS, ECFP, KRFP). We used the default hyperparameters for all models. We standardized the molecular descriptors to a mean of zero and unit variance for the SVM, MLP, and LR models. We performed a 5-fold cross-validated grid search on the training dataset to tune hyperparameters for the XGB using molecular descriptors and RF using ECFP, for both outcomes. The test dataset was not used during model training or tuning and was reserved exclusively for final evaluation. Model performance was assessed and compared using weighted and class-specific precision, recall and F1 score.

### 2.5 Y-randomization

As an additional validation step for the models, we performed a y-randomization (Rücker et al., 2007). Here, the outcome variable (y) is shuffled while maintaining equal instance counts per class. Thus, any true connection between the descriptors and the outcome is obliterated. Under these conditions, model performance is expected to decrease to chance level confirming the absence of predictive signal in the randomized data. This demonstrates that the original model performance was not driven by random correlations but by a true connection between the descriptors and the outcome. While flexible models may still achieve moderate performance on the training dataset due to overfitting of noise, generalization performance on the independent test dataset should collapse after y-randomization. For this validation, we used the optimal outcome definition and the best-performing model (XGB) from the previous experiments. We compared the weighted and class specific performance on the training and test datasets.

### 2.6 Explainable artificial intelligence

Except for decision trees (Schöning and Hammann, 2018), humans often struggle to interpret ML models due to the complexity of their underlying algorithms and decision-making processes. Explainable Artificial Intelligence (xAI) provides methods to improve the transparency and interpretability of model predictions, ensuring they are driven by meaningful patterns rather than data noise. In this study, we employed two complementary xAI approaches to interpret model behavior, selected based on the type of molecular predictors used: molecular descriptors or molecular fingerprints. Molecular descriptors are engineered molecular features that describe a molecule’s physicochemical properties and are represented by numerical, mostly continuous values. Therefore, a feature-based approach is often the most appropriate choice. We used SHAP (Shapley Additive exPlanations) to identify the most informative attributes for predicting affinity and potency using the XGB model (Molnar, 2025; Lundberg and Lee, 2017). SHAP summary and beeswarm plots were used to provide a global interpretation of feature importance across the dataset, while also reflecting the distribution of local feature contributions for individual class predictions.

Molecular fingerprints encode the presence or absence of chemical structures as high-dimensional, sparse, binary vectors. They provide a computationally efficient method for handling and comparing chemical structures. However, interpreting model predictions trained with fingerprints is challenging (Molnar, 2025), as substructure notations lack intuitive meaning. Therefore, we decided to present twelve molecules, map SHAP values to their corresponding atoms, and display their influence as heatmaps, as introduced by Feldmann and Bajorath (2022). These visualizations provide compound-specific insights into model predictions but are not intended to represent global interpretation across the dataset. We based the explanation of important atoms and substructures on ECFP, using RF models for affinity and potency. To increase comparability between molecules and improve visual differentiation in low-value regions, we normalized the maximum color intensity to 70% of the maximum SHAP value (Harren et al., 2022). Red is used in these maps to reflect a positive influence on the prediction outcome *high-affinity* or *high-potency*, and blue a negative influence. For this analysis, we decided to use JWH-018 (1-naphthalenyl (1-pentyl-1H-indol-3-yl)-methanone), eight metabolites of JWH-018 (2-OH indole JWH-018, 4-OH indole JWH-018, 5-OH indole JWH-018, 6-OH indole JWH-018, 7-OH indole JWH-018, 4-OH pentyl JWH-018, 5-OH pentyl JWH-018, and N-pentanoic acid JWH-018), THJ-2201 ([1-(5-fluoropentyl)-1H-indazol-3-yl]-1-naphthalenyl-methanone), AM-2201 ([1-(5-fluoropentyl)-1H-indol-3-yl]-1-naphthalenyl-methanone), and THJ-018 (1-naphthalenyl (1-pentyl-1H-indazol-3-yl)-methanone). All these substances have a naphthoylindole core structure with hydroxylation at different positions and side-chain modifications (hydroxylation, carboxylation, fluorination). The similarities between these substances will allow a direct comparison of the impact of the different modifications. Additionally, *in vitro* measurements of potency (Åstrand et al., 2025) and affinity (Chimalakonda et al., 2012; Brents et al., 2011) for several of these substances are available, allowing us to directly compare the predictions of our model with experimental data.

### 2.7 Correlation analysis

In addition to features contributing to the affinity or potency of substances at CB_1_, we also wanted to analyze how those two properties are related. We filtered the dataset for the largest fragment of each substance, for which affinity and potency information were present. That information did not need to come from the same study. We used the median value across all experiments if more than one result was present in the dataset. All values were capped at 10,000 nM.

### 2.8 Software

The study was conducted using Python Programming Language, version 3.11.13 (Rossum and Drake, 2009). We used RDKit, version 2024.09, to calculate ECFP descriptors. We calculated PaDEL molecular descriptors (Yap, 2011), MACCS, and KRFP using padelpy, version 0.1.16. Machine learning, including the RF, SVM, and LR models, was performed using scikit-learn, version 1.5.2 (Pedregosa et al., 2011). The XGB model was trained with xgboost, version 2.1.3. We calculated SHAP values (Lundberg and Lee, 2017) with shap, version 0.48.0. For mapping SHAP values onto molecular structures, we used MolPipeline, version 0.11.0 (Sieg et al., 2024).

## 3 Results

### 3.1 Datasets

The affinity dataset contained 7,727 instances of 6,380 (82.6%) unique substances. The vast majority of experiments were conducted with a competitive radioligand assay (99.5%) with [^3^H]CP-55940 (76%) [^3^H]SR-141716 (17%), and [^3^H]WIN-55212–2 (2%) being the most common radioligands. The most common receptor species were human (75%), rat (21%), and mouse (4%), and the most common cell membranes were HEK (human embryonic kidney) cell lines (26%), various brain tissues (24%), and CHO (Chinese hamster ovaries) cell lines (19%). The mean pKi value (negative logarithm of the Ki) and standard deviation were 6.58 ± 1.32 (Supplementary Figure S1). We subsequently excluded all experiments that did not use a competitive radioligand assay.

The potency dataset contained 5,405 instances of 4,283 (79.3%) unique substances. Potency was most commonly measured with various cAMP (58%) and [^35^S^]^GTPγS (11%) assays, using fluorescence (55%) and radioactivity (13%) as the most frequent assay readouts. The vast majority of experiments used human receptors (90%), followed by rat (5%) and mouse (1%). The most common cell membranes were CHO (58%) and HEK cell lines (23%). The mean pEC_50_ value (negative logarithm of the EC_50_) and standard deviation were 6.12 ± 1.36 (Supplementary Figure S2).

As comparisons of rat, mouse, and human CB1 sequences showed extensive homology (>96%) (Gérard et al., 1991; Chakrabarti et al., 1995), species origin was not considered as a separate covariate in this study, as the high sequence similarity suggests largely comparable ligand binding characteristics across these species.

### 3.2 Outcome engineering

We compared different class definitions (data not shown) during the development of the affinity and the potency multi-class models.

For the affinity model, we decided on the following definitions: (i) *high-affinity* (Ki < 10 nM), (ii) *low-affinity* (1,000 nM > Ki ≥ 100 nM), and (iii) *no-affinity* (Ki > 4,000 nM). Substances, where the outcome was defined as “unspecified” or “inconclusive” in PubChem, were only considered if a measured value or a larger than value (e.g., > 10,000 nM) was available. The class was then determined based on the value. This resulted in an imbalanced dataset of 4,163 substances with approximately 20%, 28%, and 51% in the *high-affinity*, *low-affinity,* and *no-affinity* classes, respectively.

For the potency model, we decided on the following definitions: (i) *high-potency* (EC_50_ < 50 nM), (ii) *low-potency* (2,000 nM > EC_50_ ≥ 200 nM), and (iii) *no-potency* (EC_50_ > 6,000 nM). Substances, where the outcome was defined as “unspecified” or “inconclusive” in PubChem, were excluded. Substances flagged as “inactive” in PubChem were allocated to the *no-potency* class. This resulted in an imbalanced dataset of 2,851 substances with approximately 33%, 23%, and 44% in the *high-potency*, *low-potency,* and *no-potency* classes, respectively. As the datasets were imbalanced, we used class weights during training to avoid overfitting to the majority class.

### 3.3 Comparison of machine learning algorithms and predictor data sets

For both outcomes, we considered 5 ML algorithms (XGB, RF, SVM, MLP, and LR) and four different predictor datasets (molecular descriptors, ECFP, MACCS, and KRFP). We calculated weighted and class specific precision, recall and F1 score (Supplementary Tables S1–S4). The corresponding confusion matrices are provided in Supplementary Figures S3–S7 and Supplementary Figures S9–S13.

For both outcomes, the nearest-neighbor Tanimoto similarity between test substances and the training dataset was analyzed to assess potential structural overlap. The observed similarity range (∼0.6–0.9) indicates that test substances are structurally related but not identical to the training data in the ECFP representation.

For the affinity model, the best performing predictor-algorithm combination on the test dataset was ECFP with RF. All measured weighted performance metrics (F1-score, precision, and recall) were 92% for this combination. Class specific analysis of the performance metrics revealed an overall reduced performance for the *low-affinity* class, but for the best performing models still ≥83%.

For the potency model, the best performing predictor-algorithm combinations were ECFP and KRFP with XBG, and molecular descriptors, MACCS, and ECFP with RF. The performance metrics (F1-score, precision, and recall) were ≥91% for these combinations. The *low-potency* class shows overall reduced performance metrics in the class specific analysis.

Hyperparameter tuning improved recall, precision, and F1 score for RF with ECFP about 1%, to 92%. All other models were not improved by hyperparameter tuning.

### 3.4 Y-randomization

We validated the affinity and potency model by performing a y-randomization using XGB. Performance metrics (F1 score, precision, and recall) for both models on the training and test datasets are shown in Supplementary Tables S5, S6 and the confusion matrices in Supplementary Figures S8, S14. Despite the shuffled outcome variable, the performance metrics on the training data set (precision, recall, and F1 score) ranged from 74% (with KRFP) to 98% (molecular descriptors). However, on the test data set, the metrics ranged from 43% to 53%.

### 3.5 SHAP values

**FIGURE 1:**
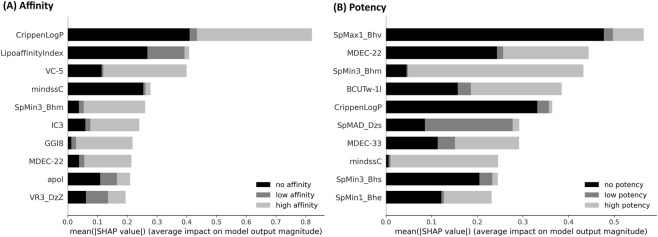
Summary plot for the overall 10 most important molecular descriptors (explanation of molecular descriptor abbreviations see Supplementary Table S7) **(A)** Affinity plot **(B)** Potency plot.

**FIGURE 2:**
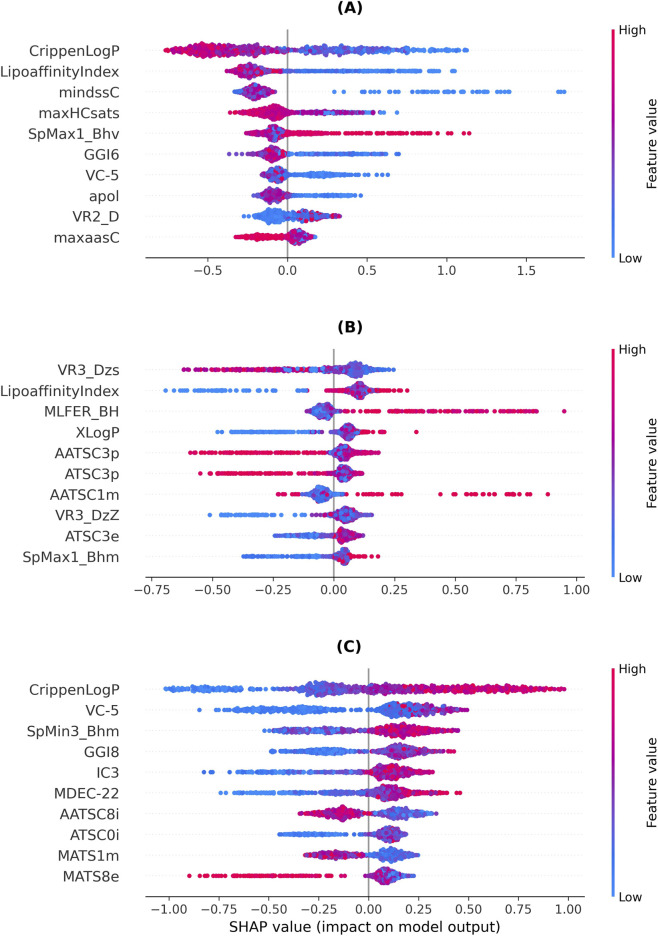
Affinity prediction: Bee-swarm plots for the ten most important features per class (explanation of molecular descriptor abbreviations see Supplementary Table S7) **(A)** No affinity **(B)** Low affinity **(C)** High affinity.

**FIGURE 3:**
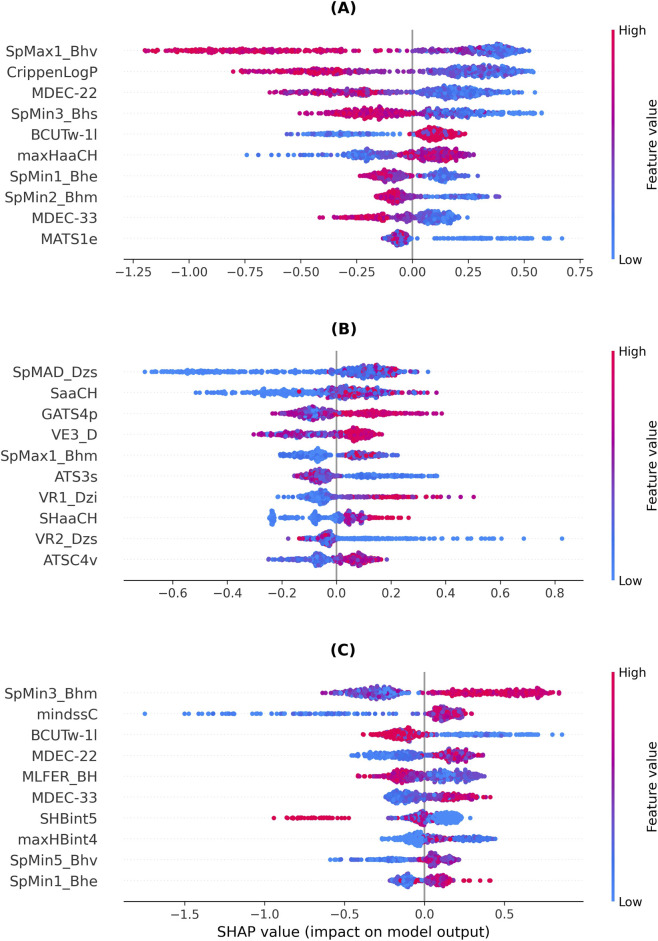
Potency prediction: Bee-swarm plots for the ten most important features per class (explanation of molecular descriptor abbreviations see Supplementary Table S7) **(A)** No potency **(B)** Low potency **(C)** High potency.

The ten most important molecular descriptors and their mean overall SHAP values for the affinity and potency models are summarized in Figure 1, whereas the bee swarm plots for affinity and potency are shown in Figures 2, 3, respectively. Further explanation of the mentioned single molecular descriptors is provided in the Supplementary Table S7.

The most important molecular descriptor to determine the binding affinity of a substance to the CB_1_ is Crippen’s LogP (CLogP), i.e., a measure of hydrophilicity. Lower CLogP values are more predictive of the *no-affinity* class, whereas higher values are more predictive of the *high-affinity* class. The second most important molecular descriptor is the lipo-affinity index, i.e., the affinity of a molecule to lipophilic environments, with low values more indicative of the *no-affinity* class.

In summary, five classes of molecular descriptors are mainly represented in the overall and class-specific top-10 molecular descriptors for affinity prediction: (i) Atom type electrotopological state (LipoaffinityIndex, mindssC, maxHCsats, maxwHBa), (ii) Autocorrelation (AATS6i, AATSC1m, AATSC3p, AATSC4e, ATSC0i, ATSC3e, ATSC3p, GATS8s, MATS5m), (iii) Barysz matrix eigenvectors (SpMAD_DzZ, VR3_Dzs), (iv) Burden modified eigenvalues (SpMax1_Bhv, SpMax4_Bhv, SpMin3_Bhm, SpMin4_Bhm), and (v) Topological charge (GGI7, GGI8) (Supplementary Table S7).

For potency prediction, Burden’s modified eigenvalues (Burden, 1997) (SpMax and SpMin descriptors) appear to contribute substantially to the model’s decision. Overall, low values support the prediction for the *no-potency* class, whereas higher values favor the *high-potency* class. Crippen’s LogP is the second most important descriptor for the *no-potency* class; however, it is less important for the other two classes. Additionally, the descriptor classes atom type electrotopological state (maxHaaCH, maxHBint4, mindssC, SaaCH, SHaaCH, SHBint5), autocorrelation (ATS3s, ATSC4v, GATS4p, MATS1e), Barysz matrix (SpMAD_Dzs, VE3_D, VR1_Dzi, VR2_Dzs), and molecular distance edge (MDEC-22, MDEC-33) contribute to the model’s prediction.

### 3.6 Structural heatmap of SHAP values

**FIGURE 4:**
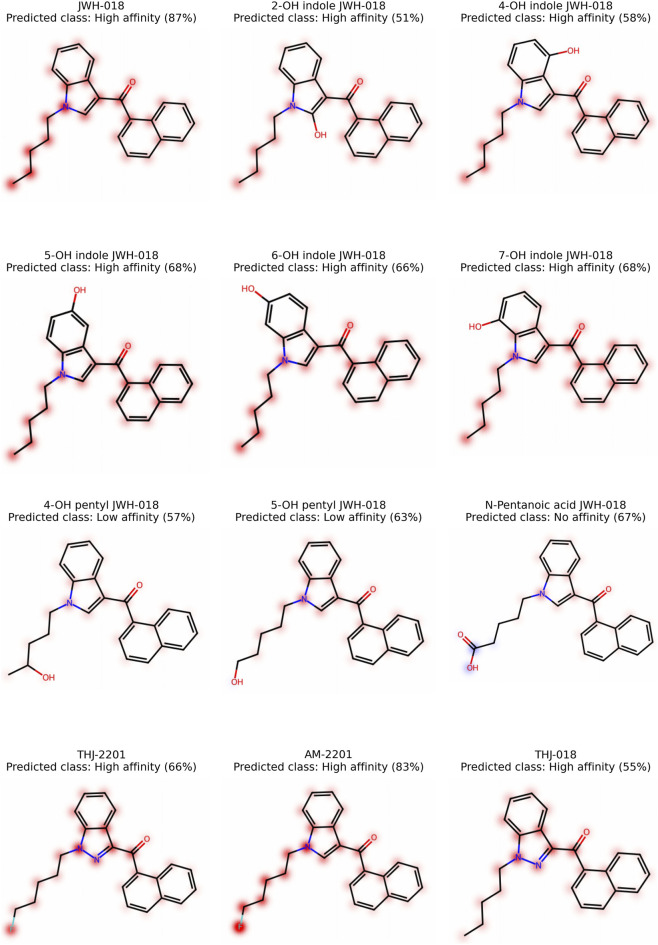
Affinity: Mapping of SHAP values to the corresponding atoms. Atoms associated with positive contributions to the *high-affinity* class by the model are highlighted in red, while atoms with negative contributions are highlighted in blue. The model probability of the predicted class is indicated in brackets.

**FIGURE 5:**
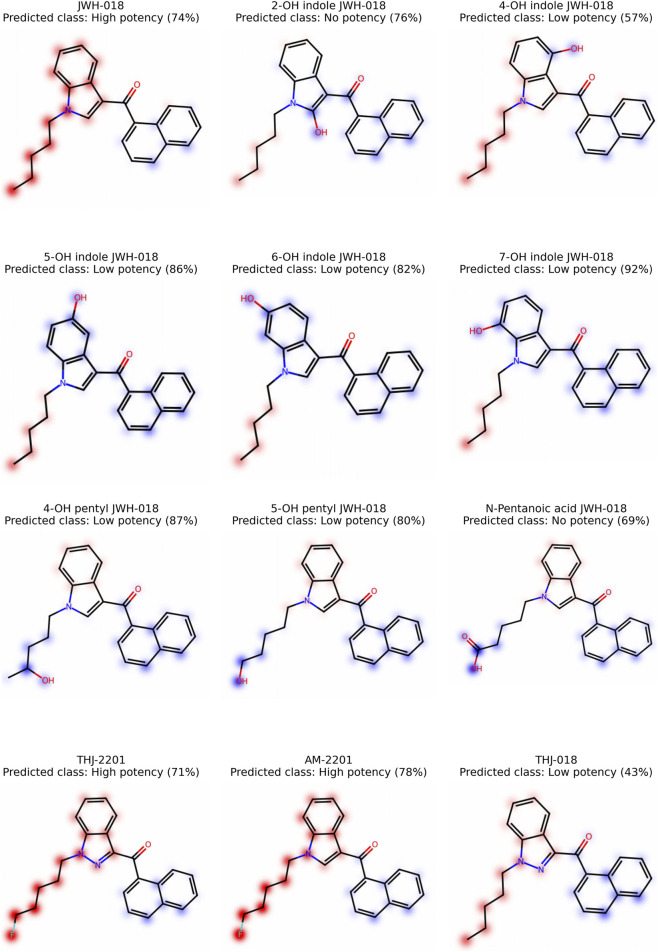
Potency: Mapping of SHAP values to the corresponding atoms. Atoms associated with positive contributions to the *high-potency* class by the model are highlighted in red, while atoms with negative contributions are highlighted in blue. The model probability of the predicted class is indicated in brackets.

We created heatmaps for JWH-018 and its eight metabolites, THJ-2201, AM-2201, and THJ-018 for the affinity (Figure 4) and the potency model (Figure 5). In the affinity model, the predicted classes of JWH-018 and its metabolites are largely in accordance with published results. The prediction of JWH 018 and 4-OH indole JWH-018 as *high-affinity*, and N-pentanoic acid JWH-018 as *no-affinity* are in accordance with experimental observations from Brents et al. (Brents et al., 2011). Experimental binding affinities of 6-OH indole JWH-018 (prediction *high-affinity*), 7-OH indole JWH-018 (prediction *high-affinity*), and 5-OH pentyl JWH-018 (prediction low-affinity) are between the *high-* and *low-affinity* class (Brents et al., 2011) and are thus difficult to interpret. The prediction of AM-2201 as *high-affinity* is supported by *in vitro* experiments (Chimalakonda et al., 2012). A detailed comparison is provided in Supplementary Table S8.

The heatmaps indicate that the pentyl tail has a positive influence on prediction as *high-affinity*. However, modification of the pentyl tail change this effect: hydroxylation or carboxylation diminishes and fluorination increases the impact. Replacement of the indole with an indazole reduces the overall positive contribution of the pentyl tail (THJ-2201 vs. AM-2201 and JWH-018 vs. THJ-018). While hydroxyindole groups still have a slightly positive contribution on affinity, this positive contribution is reduced compared to the indole without hydroxy group. The hydroxy group itself neither has a positive nor negative impact.

The predicted potency class of our potency model is in line with the experimental data from Åstrand et al. (2025) for nine of the twelve substances (JWH-018, 2-OH indole JWH-018, 5-OH indole JWH-018, 6-OH indole JWH-018, 4-OH pentyl JWH-018, 5-OH pentyl JWH-018, N-pentanoic acid JWH-018, THJ-018, and THJ-2201). N-pentanoic acid JWH-018 and 2-OH indole JWH-018 were both predicted as *no-potency*, with no EC_50_ calculated in the experimental data. The SCRA THJ-018 was misclassified as *low-potency* with low probability (<45%). However, the experimental EC_50_ was 48.80 nM, which should be within the *high-potency* class (<50 nM). The metabolite 7-OH indole JWH-018 was also predicted as *low-potency* with high probability (>90%), but no EC_50_ was calculated in the experimental data, implying a very low potency. The metabolite 4-OH indole JWH-018 had an EC_50_ of 83.58 nM, a result which is within the gap between the *high-* and *low-potency* classes of the prediction model (50–200 nM). The substance was predicted as *low-potency* with low probability (<60%). A detailed comparison is provided in Supplementary Table S8.

The heatmaps for the potency model also reveal the positive contribution of the pentyl tail to the potency of the molecule. While still positive, hydroxylation of the indole adversely affects the contribution of the pentyl tail. Hydroxylation and carboxylation of the pentyl tail, on the other hand, resulted in a negative impact on potency. Fluorination increases the positive effect of the pentyl tail on potency. The hydroxy group of the hydroxyindole adversely influences the potency. In all analyzed molecules, the naphthalene group showed a slightly negative contribution to the prediction of the *high-potency* class, indicating that, within the context of the model, its presence is less associated with the most potent compounds compared to other structural features.

### 3.7 Correlation analysis

We identified 247 substances with available affinity and potency data. A Spearman’s correlation was used to determine the relationship between those values. We identified a moderate, positive monotonic correlation between affinity and potency (ρ = 0.63, confidence interval 0.55–0.70, p < 0.001, see Supplementary Figure S15) (Schober et al., 2018).

## 4 Discussion

In this study, we compiled a dataset of substances with experimentally measured CB_1_ binding affinity and functional potency. We trained and compared five multi-class ML algorithms using four types of molecular descriptors and fingerprints for each endpoint. We used hold-out validation and y-randomization to assess the validity of our models. Lastly, we calculated SHAP values to explain the features’ contribution to the model prediction. Especially XGB with molecular descriptor and RF with ECFP, outperformed the other models for the prediction of binding affinity (recall, precision, and F1 score >90%). In terms of potency, XGB and RF showed excellent performance with either molecular descriptors or ECFP (recall, precision, and F1 score >90%). Noteworthy are the results from y-randomization. Even though the outcomes were randomized and thus unrelated to the predictors, the performance on the training datasets reached precision, recall, and F1 scores of up to 98%. However, on the test dataset, the performance dropped to approximately 50%. Thus, we can rule out chance correlations in our actual models, as those would show up as a pronounced drop in the performance on the test dataset, as apparent in the y-randomization.

To provide insight into the model’s classification process and to compare molecular properties that drive affinity and potency, we calculated SHAP values. Overall, descriptors related to the lipophilicity of a ligand are important properties driving its binding affinity. These traits determine the ligand’s ability to partition into and migrate within the membrane environment that leads to the receptor’s binding site. The CB_1_, a GPCR, is a membrane-embedded functional protein. To reach their binding site, lipophilic or amphiphilic ligands first embed themselves in the cell membrane. Within the membrane, the ligands align in a preferred orientation and location, determined by their electronic and stereochemical features, and then undergo fast lateral diffusion to reach the binding site (Makriyannis et al., 2005). Ligand orientation influences receptor recognition and promotes collision with the respective binding site, thereby altering binding affinity (Szlenk et al., 2019). Lipophilicity is captured in our model directly through parameters such as CLogP, but also indirectly by descriptors such as atom-type electrotopological states (e.g., lipoaffinity), mass- and polarizability-weighted autocorrelation descriptors, and topological charge indices describing the spatial distribution of polarity.

On the other hand, ligand properties driving functional potency reflect a broader, more complex structure-activity relationship. Lipophilicity of ligand was retained as CLogP, atom-type electro-topological, and autocorrelation descriptors, states weighted by polarizabilities or I-state. Interesting to note here is that CLogP is important for identifying *non-potent* compounds, but was secondary for differentiating between low and high potency. In contrast to the affinity model, descriptors related to molecular shape and branching features influence potency model predictions to a greater extent. For example, Burden modified eigenvalues are defined as the eigenvalues of a modified adjacency (Burden) matrix, and the lowest values (SpMin) are influenced by all atoms in the structure and therefore capture topological features of the entire molecule (Todeschini and Consonni, 2009). Further, the atom-type electrotopological state is not a pure electronic descriptor but also represents atom polarity and steric accessibility (Todeschini and Consonni, 2009). Molecular distance edge count and Barysz matrix values further indicate the importance of topological features for potency.

Taken together, while lipophilicity is necessary to reach the membrane-embedded receptor (affinity), achieving a response (potency) additionally requires the correct steric, electronic, and topological configuration to activate CB_1_. Furthermore, while affinity and potency have a positive monotonic correlation, it was only moderate, further indicating that shared and independent features drive these endpoints.

The model predictions for the substances used to create the SHAP heatmaps were consistent with *in vitro* experimental data. This result supports the use of the models for structure-activity analysis of closely related substances. The heatmaps indicate the importance of the pentyl tail and its modifications for affinity and potency. Keimowitz et al. found that the length of the side chain was directly related to receptor affinity. They hypothesized that not only the extensions of the tail away from the point of attachment, but also the folding back, bringing the terminus in the proximity of the ring system, are important pharmacophoric requirements (Keimowitz et al., 2000). An *in vitro* study showed that terminal fluorination increased potency of SCRAs (Banister et al., 2015), which also aligns with our observations.

The study has some limitations. Focusing on substances with a high affinity and/or potency such as NPS might adversely affect the performance on low and no affinity and potency substances. Our definition of the low and no affinity and potency group is in comparison to our focus group. We appreciate that some of those substances might have a measurable affinity or potency. Additionally, the introduction of gaps between the classes leads to the exclusion of compounds, thereby adversely affecting the overall chemical space. At the same time as this approach reduces ambiguity in class labels and improves class separability, it also simplifies the classification task and thus may lead to optimistic performance estimates. Mapping SHAP values of ECFP to individual atoms in a heatmap has inherent limitations that need to be considered. ECFP belong to the class of circular and topological fingerprints. They do not have predefined substructural fragments; instead, they are generated based on the chemical dataset. The calculated fragments are therefore more comprehensive and can capture more abundant and detailed structural information, including distinctive and novel substructures related to biological activity (Yang et al., 2022). Therefore, ECFP are inherently fragment-, and not atom-based (Rasmussen et al., 2023). On the other hand, mapping of SHAP values to atoms of a molecular structure omits all non-atom-localized contributions, e.g., topological patterns not uniquely assigned to an atom. Therefore, the corresponding heatmap only reflects a small window in the model reasoning. Overall, SHAP values reflect model-based, relative feature contributions and do not necessarily present a direct causal relationship between a structural feature and biological activity.

In summary, we successfully built multiple classification models to predict affinity and potency at the CB_1_ receptor, achieving excellent predictive performance. We validated the models using an external test dataset and y-randomization. The use of explainable AI, i.e., SHAP values, provided further insight into the model’s predictions and the features driving affinity and potency. The contribution of structural feature to high affinity and potency aligns with previously conducted *in vitro* experiments.

## Data Availability

Publicly available datasets were analyzed in this study. This data can be found here: https://github.com/cptbern/CB1_ML.
