# Supplementary material for: Predicting affinity and potency of new psychoactive substances at cannabinoid 1 receptor with explainable artificial intelligence
Source: Front Pharmacol. 2026 May 20;17:1815814. doi: 10.3389/fphar.2026.1815814 (PMC13229860; doi:10.3389/fphar.2026.1815814)
Supplement: Supplementary file 1 [file DataSheet1.pdf]

Supplementary Material

Predicting Affinity and Potency of New Psychoactive Substances at Cannabinoid 1 receptor with Explainable Artificial Intelligence

Supplementary Figure 1. Distribution of pKi values (negative logarithm of the Ki) of the whole data set. 3

Supplementary Figure 2. Distribution of pEC50 values (negative logarithm of the EC50) of the whole data set. 3

Supplementary Figure 3. Affinity prediction: Confusion matrices for XGBoost on the test dataset 4

Supplementary Figure 4. Affinity prediction: Confusion matrices for Random Forest on the test dataset 5

Supplementary Figure 5. Affinity prediction: Confusion matrices for SVM on the test dataset 6

Supplementary Figure 6. Affinity prediction: Confusion matrices for MLP on the test dataset 7

Supplementary Figure 7. Affinity prediction: Confusion matrices for Logistic Regression on the test dataset 8

Supplementary Figure 8. Affinity prediction: Confusion matrices for XGBoost on the test dataset after y-randomization 9

Supplementary Figure 9. Potency prediction: Confusion matrices for XGBoost on the test dataset 10

Supplementary Figure 10. Potency prediction: Confusion matrices for Random Forest on the test dataset 11

Supplementary Figure 11. Potency prediction: Confusion matrices for SVM on the test dataset 12

Supplementary Figure 12. Potency prediction: Confusion matrices for MLP on the test dataset 13

Supplementary Figure 13. Potency prediction: Confusion matrices for Logistic Regression on the test dataset 14

Supplementary Figure 14. Potency prediction: Confusion matrices for XGBoost on the test dataset after y-randomization 15

Supplementary Figure 15. Correlation between affinity and potency 16

Supplementary Table 1. Affinity prediction: Comparison of weighted performance metrics of different machine learning algorithms and predictor data sets 17

Supplementary Table 2. Affinity prediction: Comparison of class specific performance metrics of different machine learning algorithms and predictor data sets 17

Supplementary Table 3. Potency prediction: Comparison of weighted performance of different machine learning algorithms and predictor data sets 18

Supplementary Table 4. Potency prediction: Comparison of class specific performance metrics of different machine learning algorithms and predictor data sets 19

Supplementary Table 5. Affinity prediction: Weighted and class specific performance metrics after y-randomization using XGB 20

Supplementary Table 6. Potency prediction: Weighted and class specific performance metrics after y-randomization using XGB 21

Supplementary Table 7. Explanation of molecular descriptors 21

Supplementary Table 8. Comparison of model predictions with published experimental results . 23

1 Figures

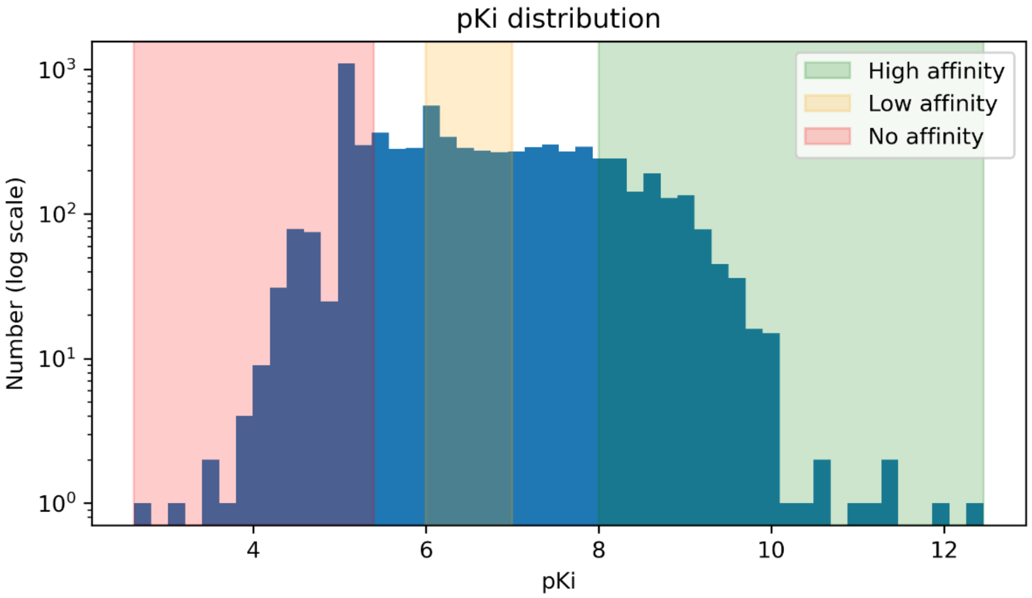

Supplementary Figure 1. Distribution of pKi values (negative logarithm of the Ki) of the whole data set.

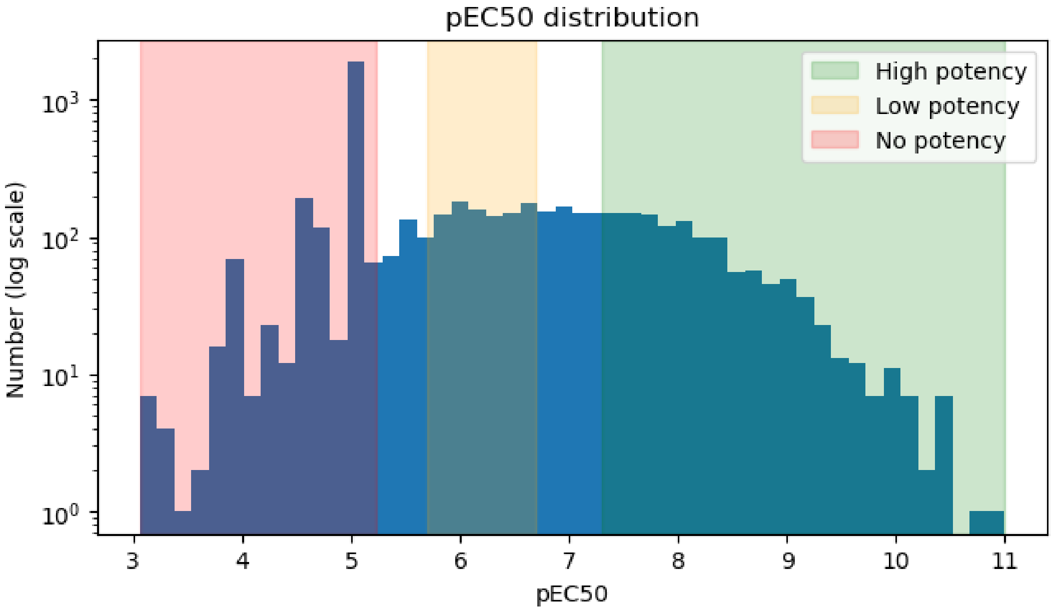

Supplementary Figure 2. Distribution of pEC50 values (negative logarithm of the EC50) of the whole data set.

A. Molecular descriptor

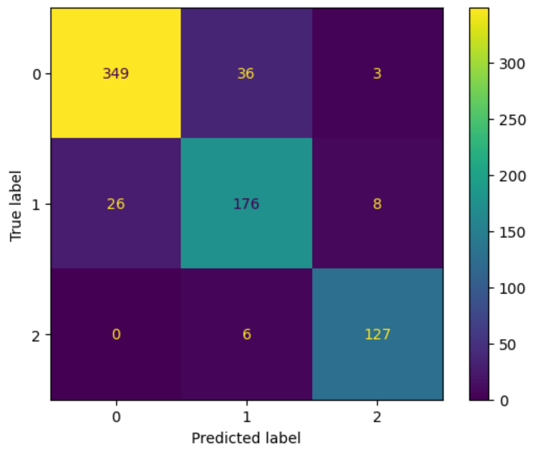

B. ECFP

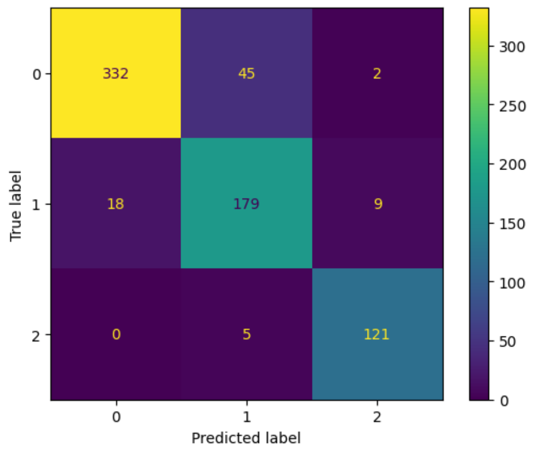

C. MACCS

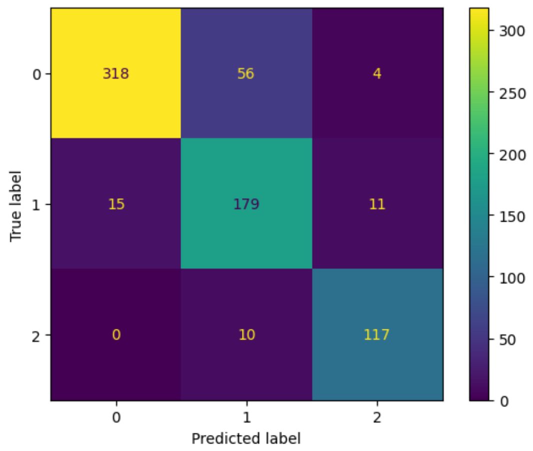

D. Klekota Roth

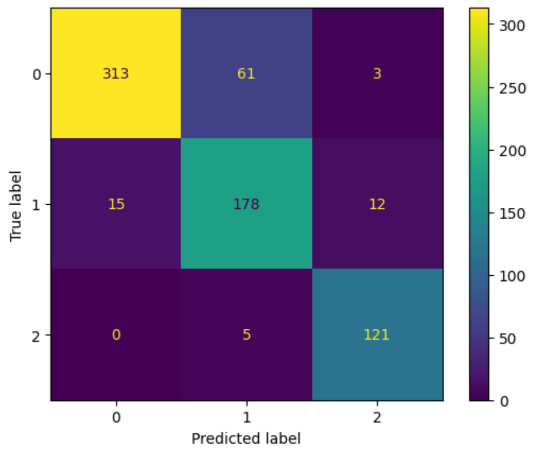

Supplementary Figure 3. Affinity prediction: Confusion matrices for XGBoost on the test dataset

A. Molecular descriptor

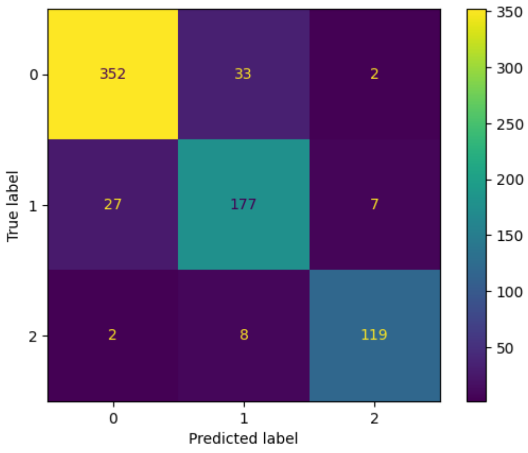

B. ECFP

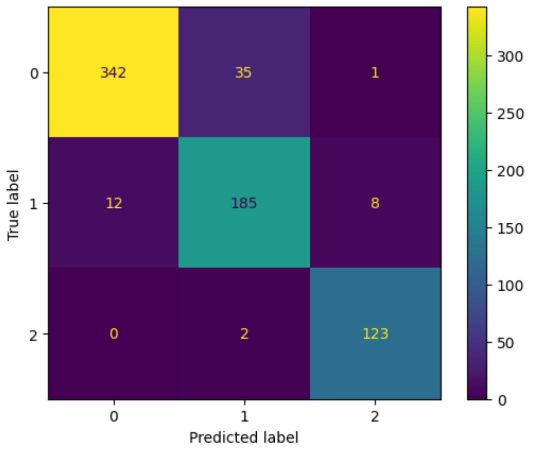

C. MACCS

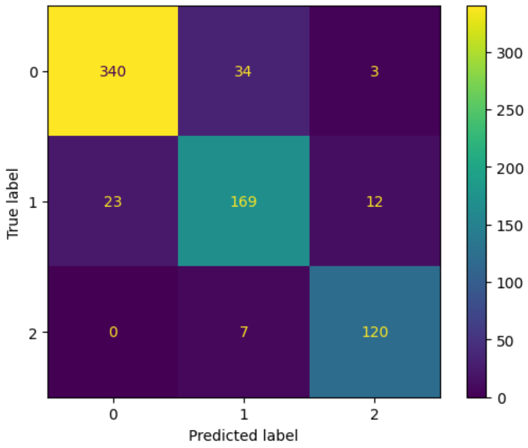

D. Klekota Roth

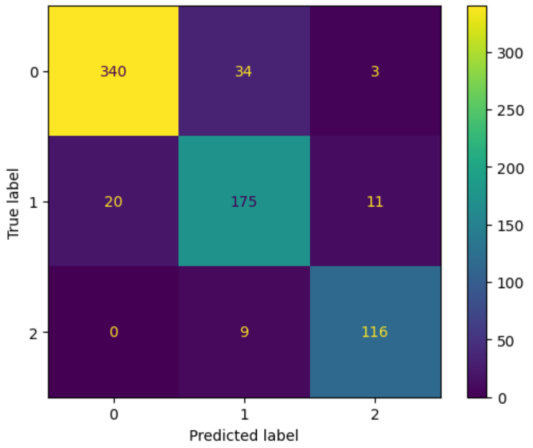

Supplementary Figure 4. Affinity prediction: Confusion matrices for Random Forest on the test dataset

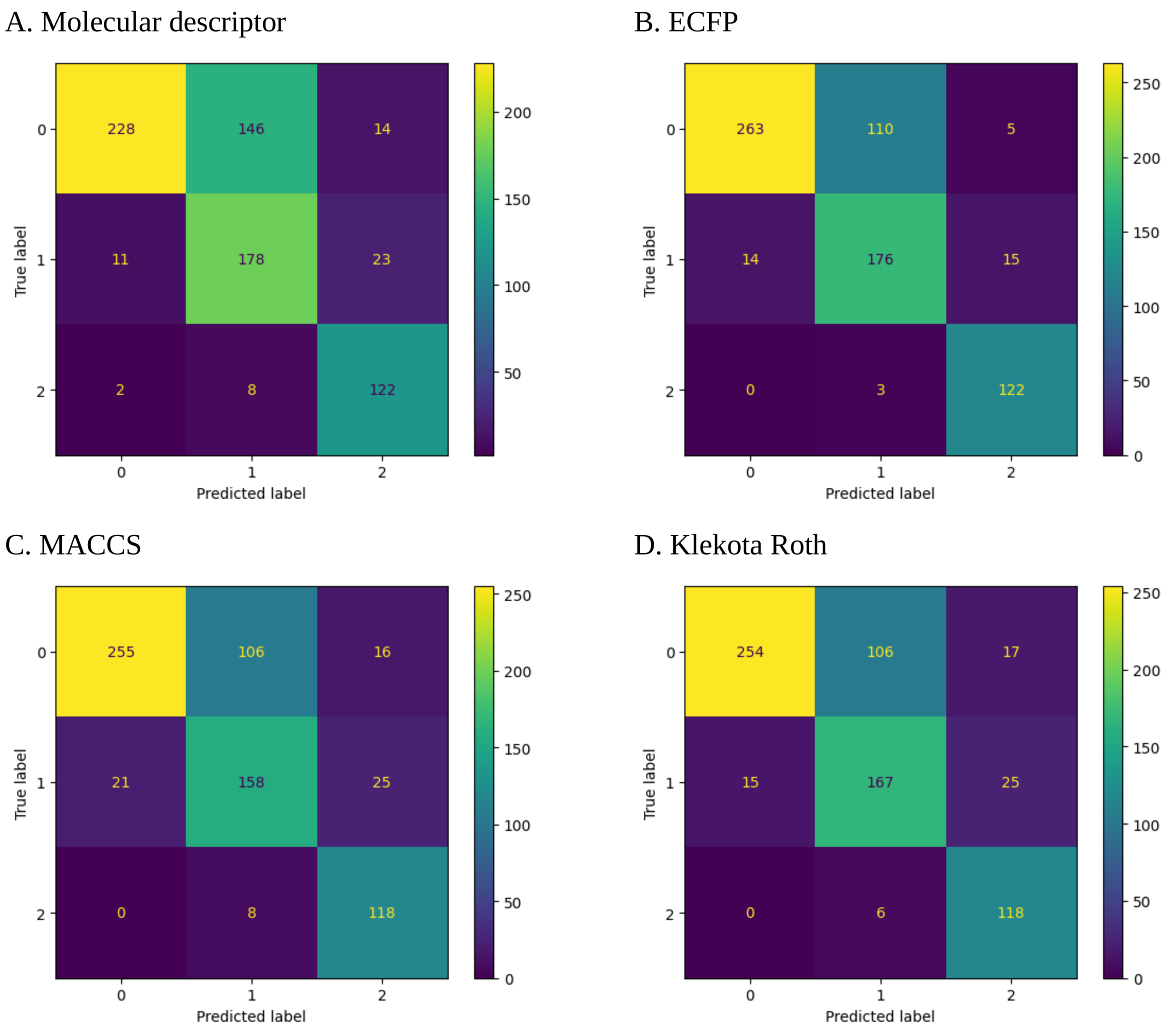

Supplementary Figure 5. Affinity prediction: Confusion matrices for SVM on the test dataset

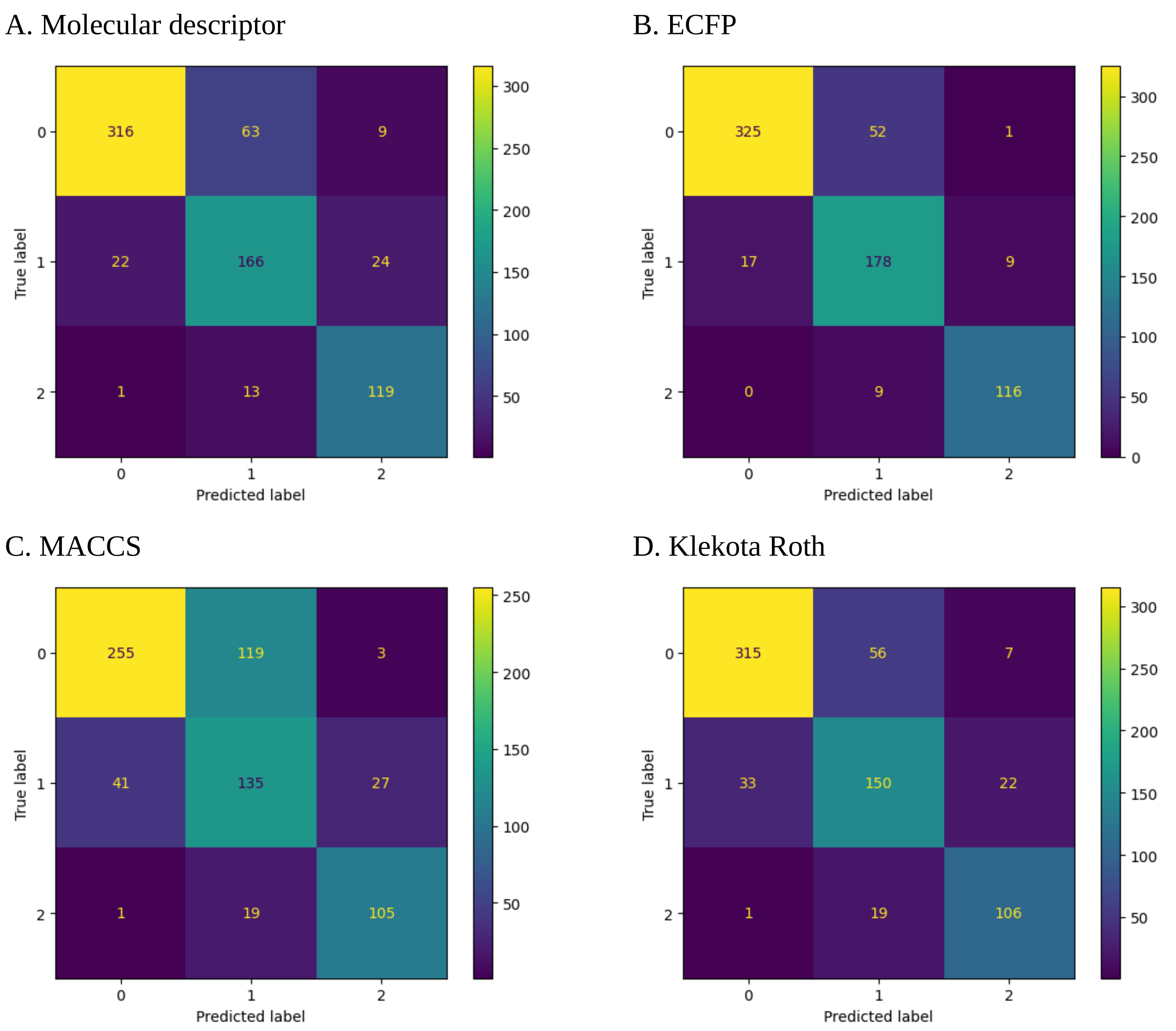

Supplementary Figure 6. Affinity prediction: Confusion matrices for MLP on the test dataset

A. Molecular descriptor

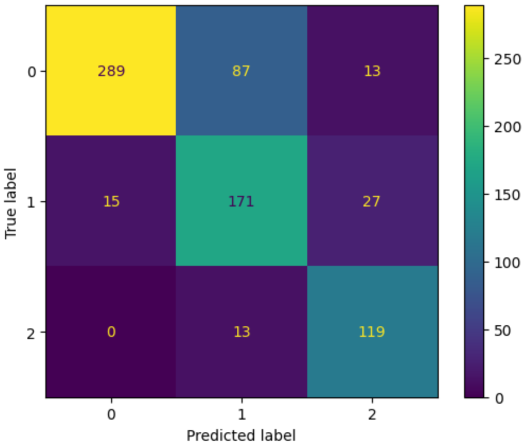

B. ECFP

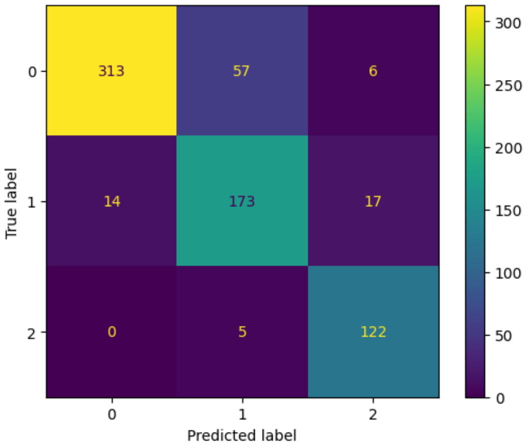

C. MACCS

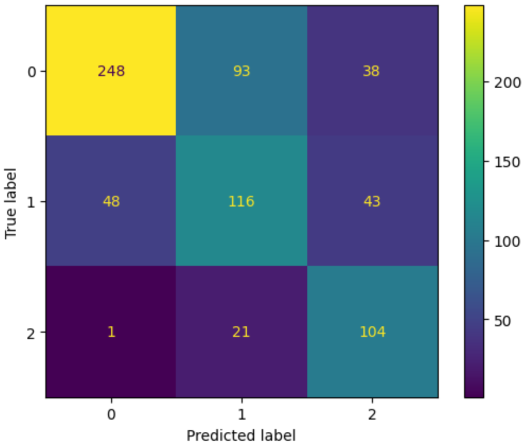

D. Klekota Roth

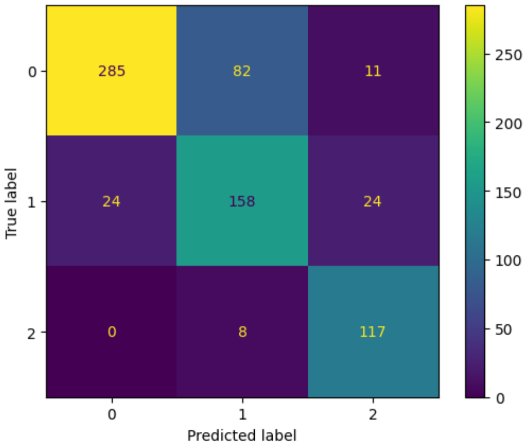

Supplementary Figure 7. Affinity prediction: Confusion matrices for Logistic Regression on the test dataset

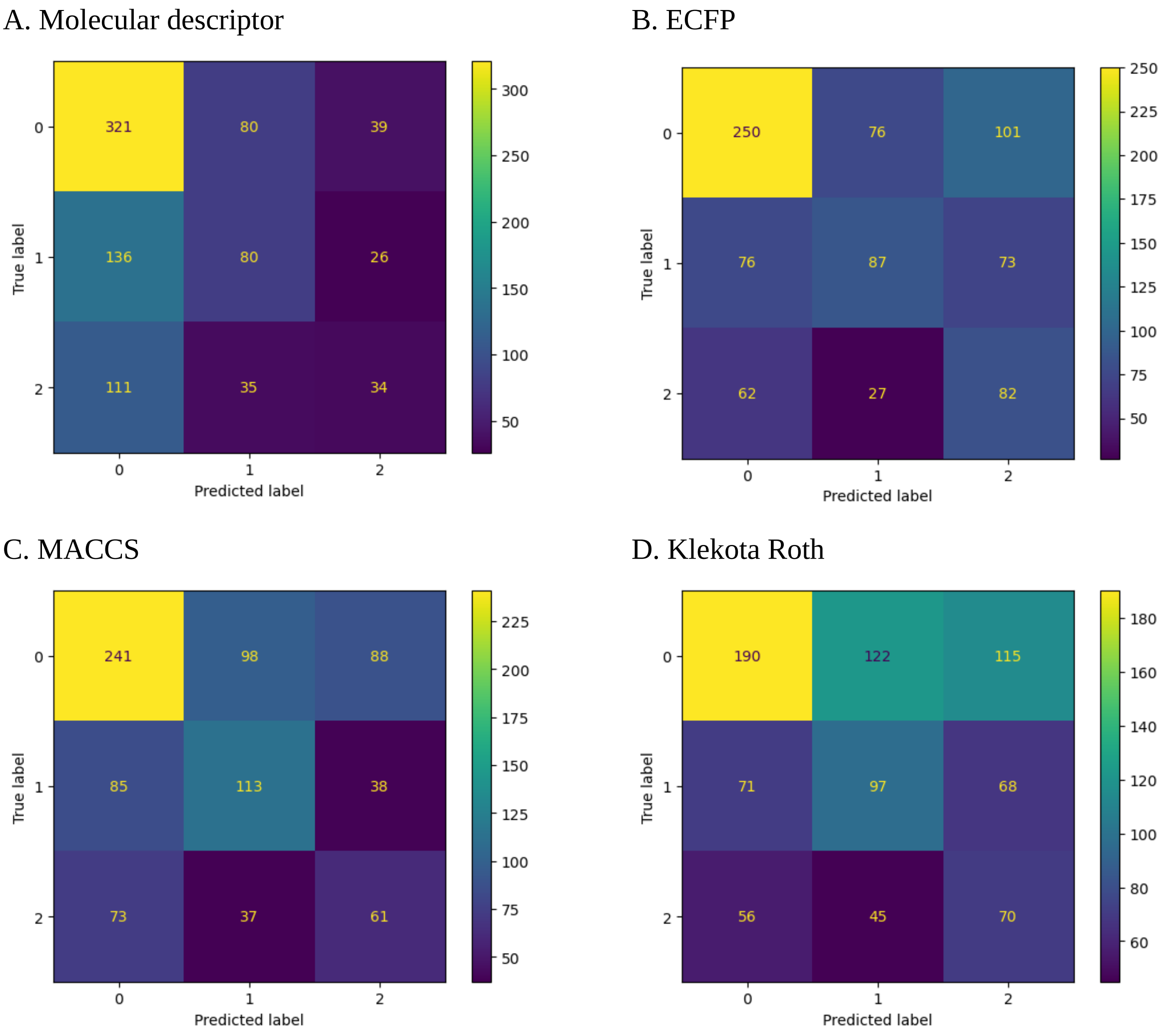

Supplementary Figure 8. Affinity prediction: Confusion matrices for XGBoost on the test dataset after y-randomization

A. Molecular descriptor

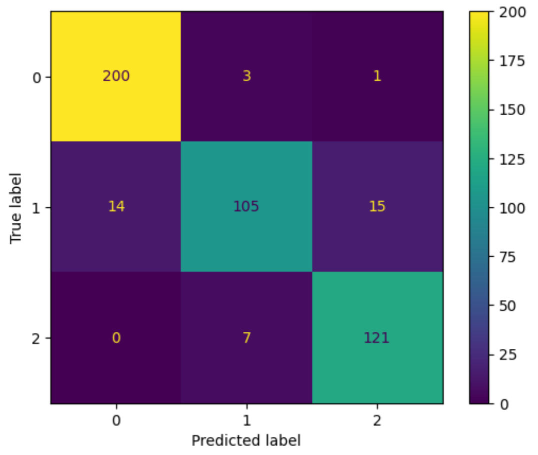

B. ECFP

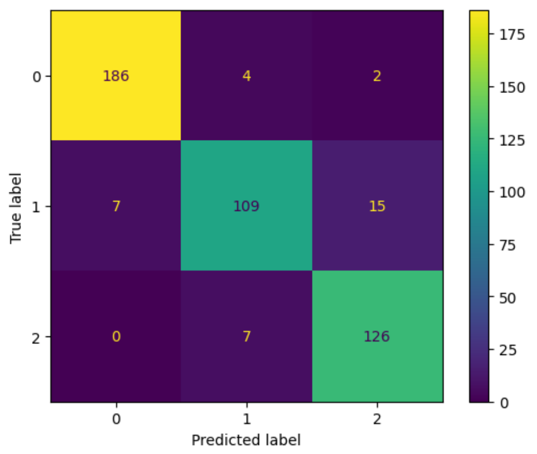

C. MACCS

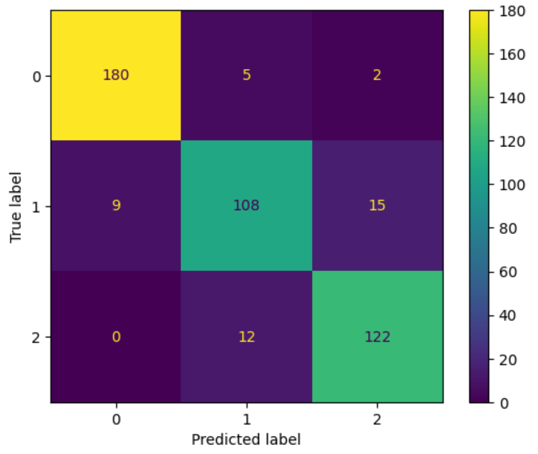

D. Klekota Roth

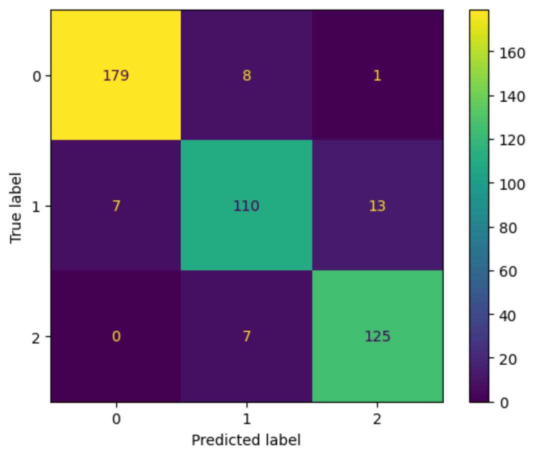

Supplementary Figure 9. Potency prediction: Confusion matrices for XGBoost on the test dataset

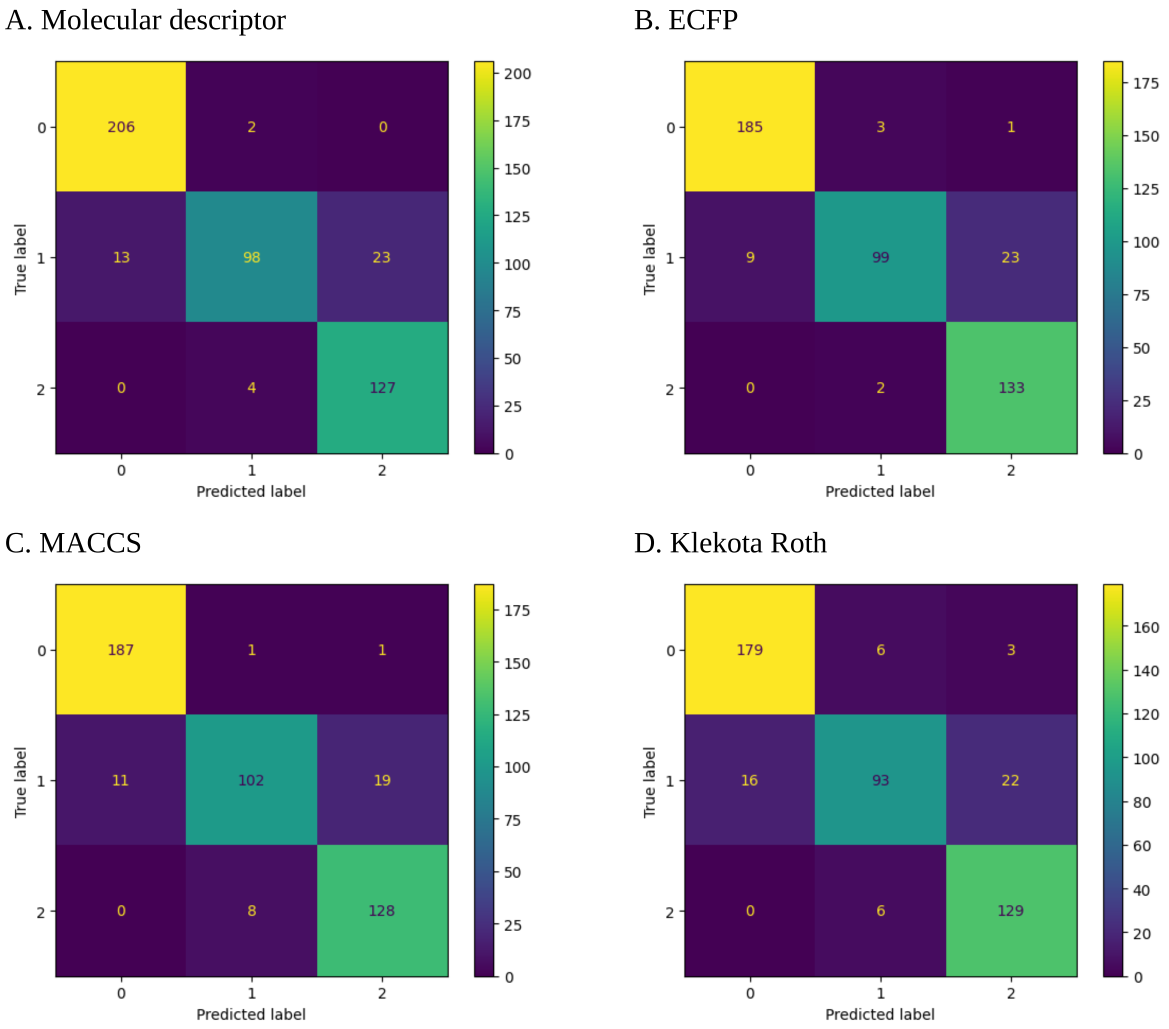

Supplementary Figure 10. Potency prediction: Confusion matrices for Random Forest on the test dataset

A. Molecular descriptor

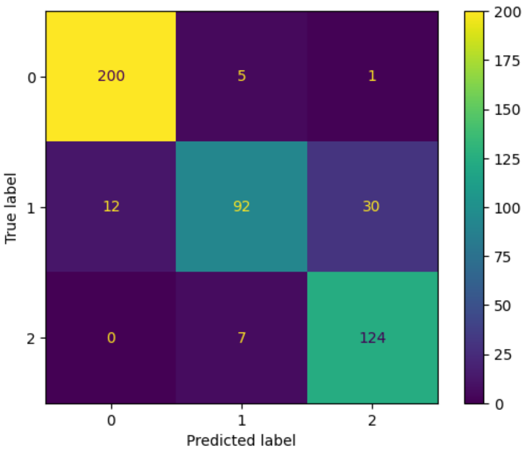

B. ECFP

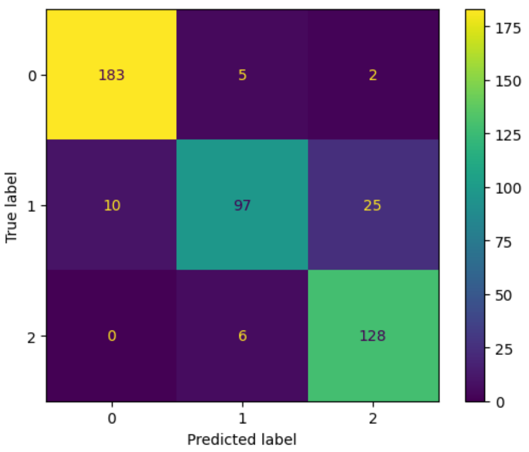

C. MACCS

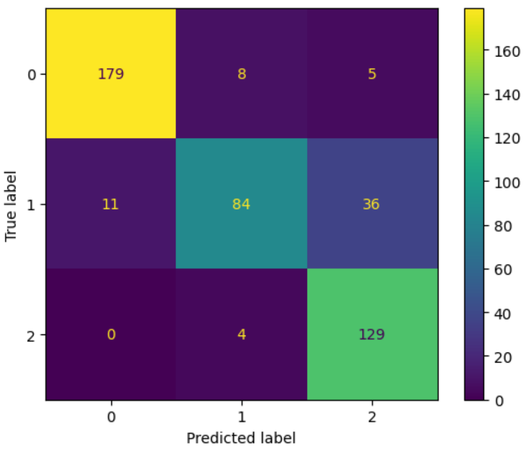

D. Klekota Roth

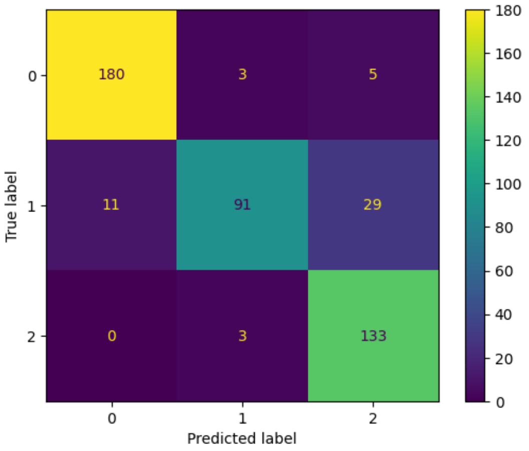

Supplementary Figure 11. Potency prediction: Confusion matrices for SVM on the test dataset

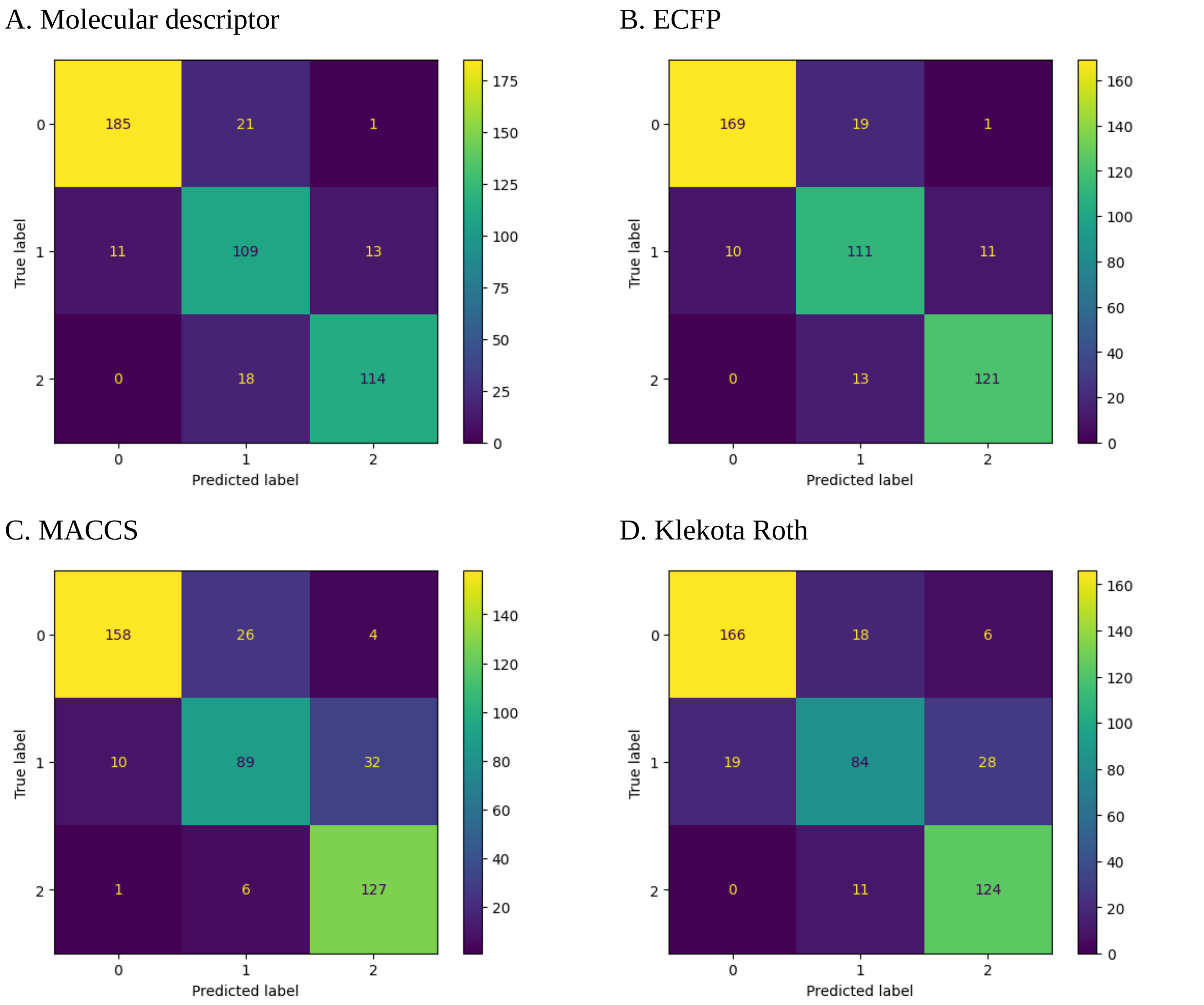

Supplementary Figure 12. Potency prediction: Confusion matrices for MLP on the test dataset

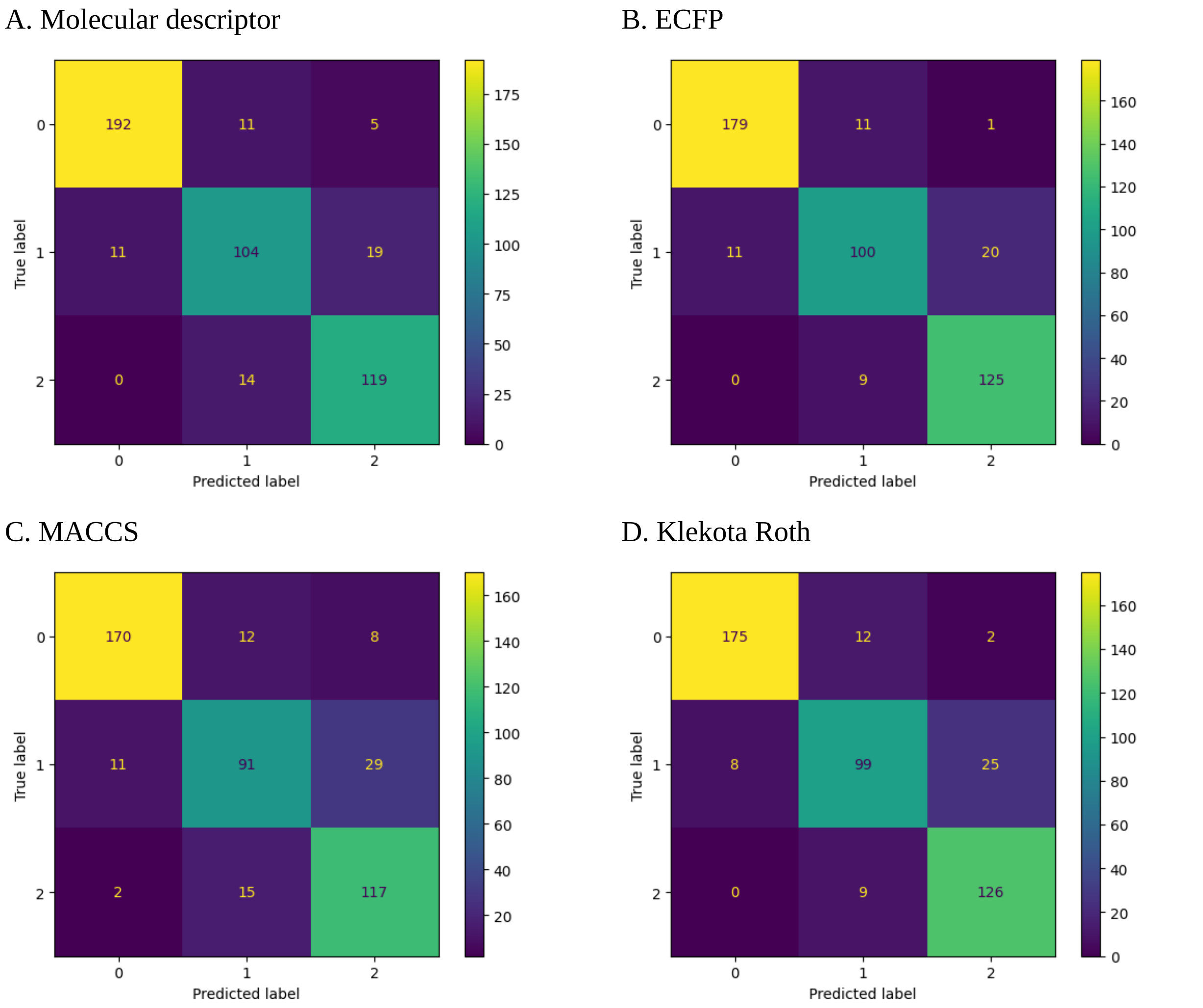

Supplementary Figure 13. Potency prediction: Confusion matrices for Logistic Regression on the test dataset

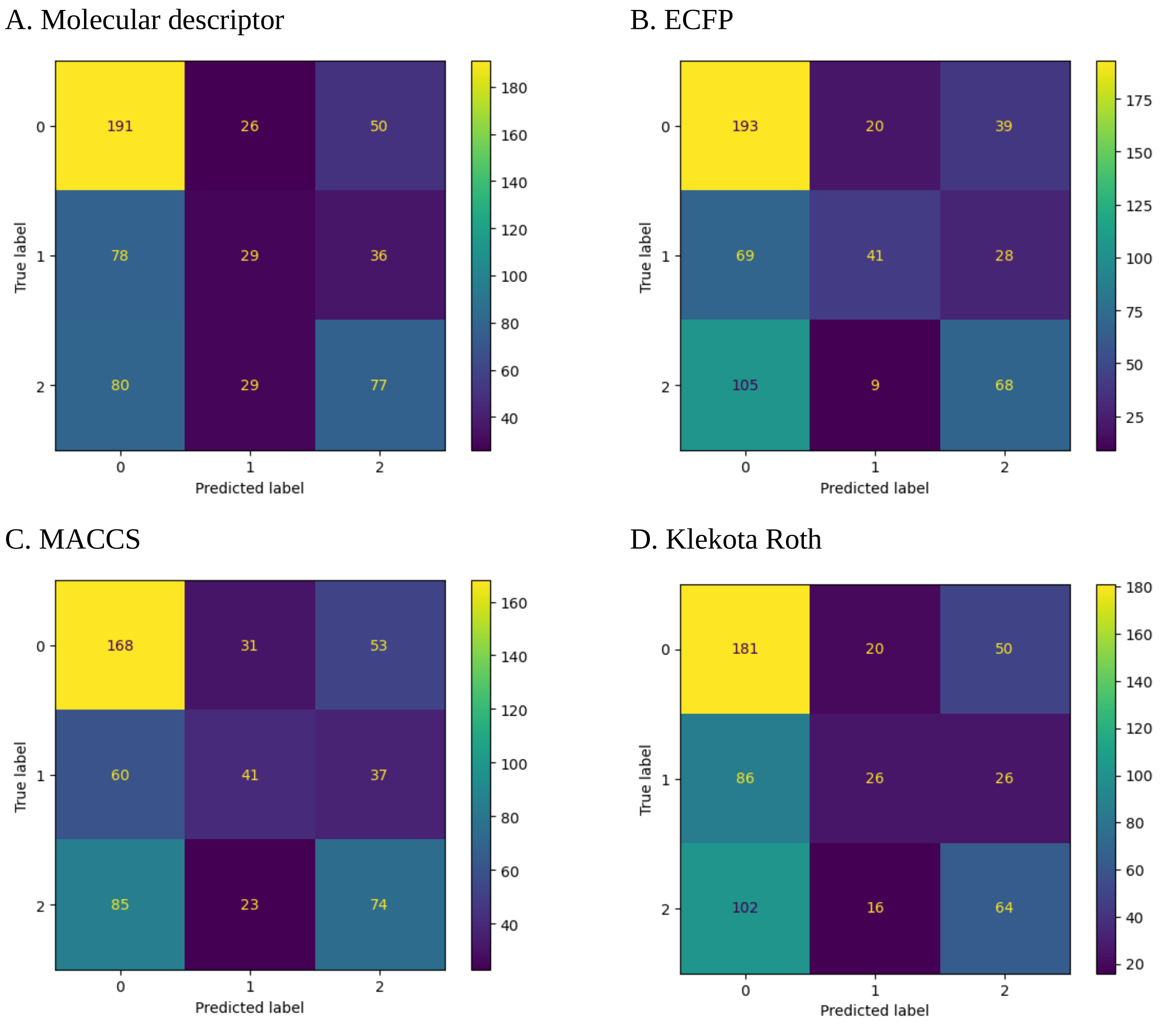

Supplementary Figure 14. Potency prediction: Confusion matrices for XGBoost on the test dataset after y-randomization

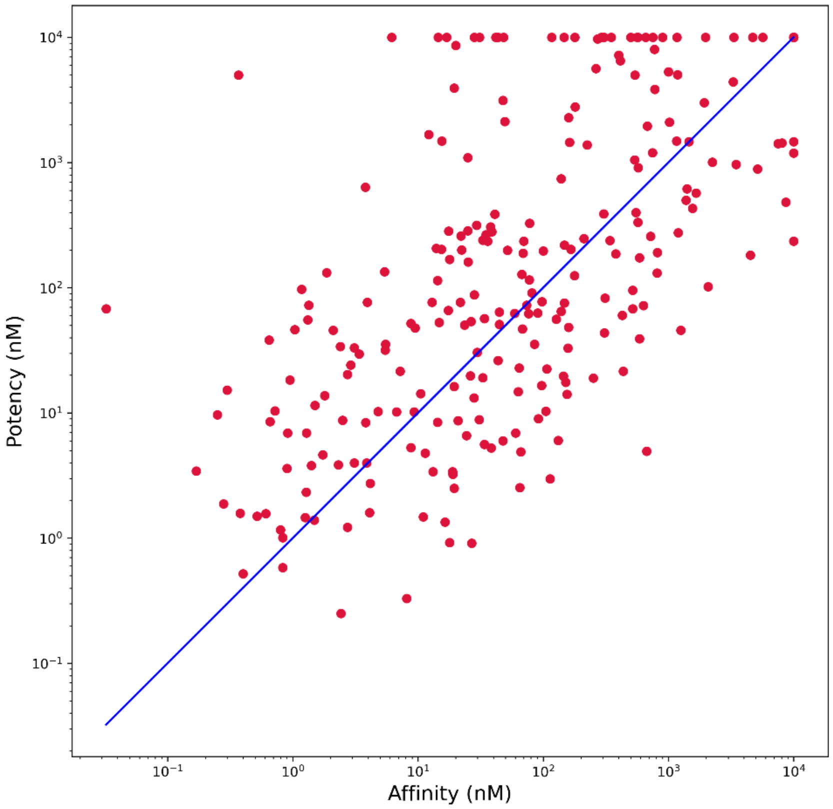

Supplementary Figure 15. Correlation between affinity and potency

Supplementary Table 1. Affinity prediction: Comparison of weighted performance metrics of different machine learning algorithms and predictor data sets

		Train			Test		
**Model**	**Predictors**	**F1**	**Precision**	**Recall**	**F1**	**Precision**	**Recall**
XGB	MolDesc	0.99	0.99	0.99	0.89	0.89	0.89
	ECFP	0.97	0.97	0.97	0.89	0.89	0.89
	MACCS	0.96	0.96	0.96	0.87	0.88	0.86
	KRFP	0.93	0.94	0.93	0.87	0.88	0.86
RF	MolDesc	0.99	0.99	0.99	0.89	0.89	0.89
	ECFP	0.99	0.99	0.99	0.92	0.92	0.92
	MACCS	0.98	0.98	0.98	0.89	0.89	0.89
	KRFP	0.99	0.99	0.99	0.89	0.89	0.89
SVM	MolDesc	0.83	0.85	0.83	0.73	0.8	0.72
	ECFP	0.9	0.9	0.89	0.8	0.83	0.79
	MACCS	0.8	0.81	0.79	0.76	0.79	0.75
	KRFP	0.84	0.85	0.83	0.77	0.81	0.76
MLP	MolDesc	0.96	0.96	0.96	0.82	0.83	0.82
	ECFP	0.98	0.98	0.98	0.88	0.89	0.88
	MACCS	0.85	0.85	0.84	0.71	0.74	0.7
	KRFP	0.97	0.97	0.97	0.81	0.81	0.81
LR	MolDesc	0.84	0.85	0.84	0.79	0.82	0.79
	ECFP	0.98	0.98	0.98	0.86	0.87	0.86
	MACCS	0.69	0.71	0.69	0.66	0.69	0.66
	KRFP	0.89	0.9	0.89	0.79	0.81	0.79

LR: Logistic Regression, MLP: Multi-Layer perceptron, MolDesc: Molecular Descriptor, RF: Random Forest, SVM: Support Vector Machine, XGB: XGBoost

Supplementary Table 2. Affinity prediction: Comparison of class specific performance metrics of different machine learning algorithms and predictor data sets

		Train [no-affinity low-affinity high-affinity]			Test [no-affinity low-affinity high-affinity]		
**Model**	**Predictors**	**F1**	**Precision**	**Recall**	**F1**	**Precision**	**Recall**
XGB	MolDesc	[1.0 0.99 0.98]	[1.0 1.0 0.97]	[1.0 0.98 1.0]	[0.91 0.82 0.94]	[0.93 0.81 0.92]	[0.90 0.84 0.95]
	ECFP	[0.98 0.95 0.98]	[1.0 0.93 0.96]	[0.96 0.97 0.99]	[0.91 0.82 0.94]	[0.95 0.78 0.92]	[0.88 0.87 0.96]
	MACCS	[0.97 0.93 0.95]	[1.0 0.91 0.93]	[0.95 0.95 0.98]	[0.89 0.80 0.90]	[0.95 0.73 0.89]	[0.84 0.87 0.92]
	KRFP	[0.95 0.89 0.94]	[0.99 0.86 0.91]	[0.92 0.92 0.97]	[0.89 0.79 0.92]	[0.95 0.73 0.89]	[0.83 0.87 0.96]
RF	MolDesc	[1.0 0.99 0.98]	[1.0 0.99 0.97]	[1.0 0.98 1.0]	[0.92 0.83 0.93]	[0.92 0.81 0.93]	[0.91 0.84 0.92]

		Train [no-affinity low-affinity high-affinity]			Test [no-affinity low-affinity high-affinity]		
**Model**	**Predictors**	**F1**	**Precision**	**Recall**	**F1**	**Precision**	**Recall**
	ECFP	[1.0 0.98 0.98]	[1.0 0.99 0.97]	[0.99 0.98 0.99]	[0.93 0.87 0.96]	[0.97 0.83 0.93]	[0.90 0.90 0.98]
	MACCS	[0.99 0.96 0.97]	[1.0 0.96 0.95]	[0.98 0.96 0.99]	[0.92 0.82 0.92]	[0.94 0.80 0.89]	[0.90 0.83 0.94]
	KRFP	[1.0 0.98 0.98]	[1.0 0.99 0.97]	[0.99 0.98 1.0]	[0.92 0.83 0.91]	[0.94 0.80 0.89]	[0.90 0.85 0.93]
SVM	MolDesc	[0.87 0.74 0.85]	[0.97 0.66 0.82]	[0.78 0.85 0.89]	[0.72 0.65 0.84]	[0.95 0.54 0.77]	[0.59 0.84 0.92]
	ECFP	[0.92 0.83 0.93]	[0.97 0.78 0.90]	[0.87 0.90 0.96]	[0.80 0.71 0.91]	[0.95 0.61 0.86]	[0.70 0.86 0.98]
	MACCS	[0.85 0.70 0.81]	[0.94 0.65 0.74]	[0.78 0.75 0.89]	[0.78 0.66 0.83]	[0.92 0.58 0.74]	[0.68 0.77 0.94]
	KRFP	[0.89 0.74 0.84]	[0.95 0.71 0.79]	[0.83 0.79 0.91]	[0.79 0.69 0.83]	[0.94 0.60 0.74]	[0.67 0.81 0.95]
MLP	MolDesc	[0.98 0.93 0.96]	[1.0 0.89 0.98]	[0.96 0.98 0.94]	[0.87 0.73 0.84]	[0.93 0.69 0.78]	[0.81 0.78 0.89]
	ECFP	[0.99 0.96 0.96]	[0.99 0.94 1.0]	[0.99 0.99 0.92]	[0.90 0.80 0.92]	[0.95 0.74 0.92]	[0.86 0.87 0.93]
	MACCS	[0.89 0.76 0.86]	[0.93 0.72 0.84]	[0.85 0.81 0.89]	[0.76 0.57 0.81]	[0.86 0.49 0.78]	[0.68 0.67 0.84]
	KRFP	[0.99 0.96 0.95]	[0.99 0.96 0.95]	[0.99 0.96 0.95]	[0.87 0.70 0.81]	[0.90 0.67 0.79]	[0.83 0.73 0.84]
LR	MolDesc	[0.89 0.76 0.85]	[0.94 0.72 0.81]	[0.84 0.81 0.89]	[0.83 0.71 0.82]	[0.95 0.63 0.75]	[0.74 0.80 0.90]
	ECFP	[0.99 0.96 0.97]	[1.0 0.96 0.96]	[0.98 0.97 0.99]	[0.89 0.79 0.90]	[0.96 0.74 0.84]	[0.83 0.85 0.96]
	MACCS	[0.78 0.55 0.69]	[0.86 0.52 0.62]	[0.71 0.58 0.79]	[0.73 0.53 0.67]	[0.84 0.50 0.56]	[0.65 0.56 0.83]
	KRFP	[0.93 0.83 0.90]	[0.97 0.80 0.86]	[0.89 0.86 0.94]	[0.83 0.70 0.84]	[0.92 0.64 0.77]	[0.75 0.77 0.94]

LR: Logistic Regression, MLP: Multi-Layer perceptron, MolDesc: Molecular Descriptor, RF: Random Forest, SVM: Support Vector Machine, XGB: XGBoost

Supplementary Table 3. Potency prediction: Comparison of weighted performance of different machine learning algorithms and predictor data sets

		Train			Test		
**Model**	**Predictors**	**F1**	**Precision**	**Recall**	**F1**	**Precision**	**Recall**
XGB	MolDesc	0.99	1.0	0.99	0.91	0.91	0.91
	ECFP	0.98	0.98	0.98	0.92	0.92	0.92
	MACCS	0.98	0.98	0.98	0.90	0.90	0.91
	KRFP	0.95	0.95	0.95	0.92	0.92	0.92
RF	MolDesc	0.99	0.99	0.99	0.91	0.92	0.91
	ECFP	0.99	0.99	0.99	0.91	0.92	0.92
	MACCS	0.98	0.98	0.98	0.91	0.91	0.91

Model	Predictors	Train			Test		
		F1	Precision	Recall	F1	Precision	Recall
	KRFP	0.99	0.99	0.99	0.88	0.88	0.88
SVM	MolDesc	0.85	0.85	0.85	0.88	0.89	0.88
	ECFP	0.88	0.88	0.88	0.89	0.9	0.89
	MACCS	0.81	0.81	0.81	0.86	0.87	0.86
	KRFP	0.85	0.85	0.85	0.88	0.9	0.89
MLP	MolDesc	0.95	0.96	0.95	0.87	0.87	0.86
	ECFP	0.98	0.98	0.98	0.88	0.89	0.88
	MACCS	0.84	0.84	0.84	0.82	0.83	0.83
	KRFP	0.97	0.98	0.97	0.82	0.82	0.82
LR	MolDesc	0.87	0.87	0.87	0.87	0.87	0.87
	ECFP	0.98	0.98	0.98	0.88	0.89	0.89
	MACCS	0.76	0.76	0.76	0.83	0.83	0.83
	KRFP	0.91	0.91	0.91	0.88	0.88	0.88

LR: Logistic Regression, MLP: Multi-Layer perceptron, MolDesc: Molecular Descriptor, RF: Random Forest, SVM: Support Vector Machine, XGB: XGBoost

Supplementary Table 4. Potency prediction: Comparison of class specific performance metrics of different machine learning algorithms and predictor data sets

Model	Predictors	Train [no-affinity low-affinity high-affinity]			Test [no-affinity low-affinity high-affinity]		
		F1	Precision	Recall	F1	Precision	Recall
XGB	MolDesc	[1.0 0.99 0.99]	[1.0 0.98 1.0]	[0.99 1.0 0.99]	[0.96 0.84 0.91]	[0.93 0.91 0.88]	[0.98 0.78 0.95]
	ECFP	[0.99 0.97 0.98]	[0.98 0.97 0.98]	[0.99 0.97 0.97]	[0.97 0.87 0.91]	[0.96 0.91 0.88]	[0.97 0.83 0.95]
	MACCS	[0.99 0.96 0.98]	[0.99 0.95 0.99]	[0.98 0.98 0.98]	[0.96 0.84 0.89]	[0.95 0.86 0.88]	[0.96 0.82 0.91]
	KRFP	[0.97 0.92 0.96]	[0.96 0.91 0.97]	[0.97 0.92 0.95]	[0.96 0.86 0.92]	[0.96 0.88 0.90]	[0.95 0.85 0.95]
RF	MolDesc	[1.0 0.99 0.99]	[1.0 0.99 0.99]	[1.0 0.99 0.99]	[0.96 0.82 0.90]	[0.94 0.94 0.85]	[0.99 0.73 0.97]
	ECFP	[1.0 0.99 0.99]	[1.0 0.99 0.99]	[1.0 0.99 1.0]	[0.97 0.84 0.91]	[0.95 0.95 0.85]	[0.98 0.76 0.99]
	MACCS	[0.99 0.97 0.98]	[0.99 0.96 0.99]	[0.99 0.98 0.98]	[0.97 0.84 0.90]	[0.94 0.92 0.86]	[0.99 0.77 0.94]
	KRFP	[1.0 0.99 0.99]	[1.0 0.99 0.99]	[1.0 0.99 0.99]	[0.93 0.79 0.89]	[0.92 0.89 0.84]	[0.95 0.71 0.96]
SVM	MolDesc	[0.91 0.72 0.86]	[0.91 0.74 0.84]	[0.90 0.70 0.88]	[0.96 0.77 0.87]	[0.94 0.88 0.80]	[0.97 0.69 0.95]
	ECFP	[0.91 0.79 0.90]	[0.90 0.79 0.90]	[0.91 0.78 0.90]	[0.96 0.81 0.89]	[0.95 0.90 0.83]	[0.96 0.73 0.96]
	MACCS	[0.88 0.66 0.82]	[0.89 0.70 0.78]	[0.87 0.62 0.87]	[0.94 0.74 0.85]	[0.94 0.88 0.76]	[0.93 0.64 0.97]

		Train [no-affinity low-affinity high-affinity]			Test [no-affinity low-affinity high-affinity]		
**Model**	**Predictors**	**F1**	**Precision**	**Recall**	**F1**	**Precision**	**Recall**
	KRFP	[0.90 0.73 0.87]	[0.90 0.76 0.85]	[0.90 0.70 0.89]	[0.95 0.80 0.88]	[0.94 0.94 0.80]	[0.96 0.69 0.98]
MLP	MolDesc	[0.97 0.90 0.96]	[1.0 0.82 1.0]	[0.94 1.0 0.92]	[0.92 0.78 0.88]	[0.94 0.74 0.89]	[0.89 0.82 0.86]
	ECFP	[0.99 0.96 0.98]	[1.0 0.93 1.0]	[0.99 1.0 0.96]	[0.92 0.81 0.91]	[0.94 0.78 0.91]	[0.89 0.84 0.90]
	MACCS	[0.89 0.70 0.88]	[0.93 0.68 0.85]	[0.86 0.71 0.91]	[0.89 0.71 0.86]	[0.93 0.74 0.78]	[0.84 0.68 0.95]
	KRFP	[0.99 0.96 0.97]	[1.0 0.99 0.94]	[0.99 0.92 1.0]	[0.89 0.69 0.85]	[0.90 0.74 0.78]	[0.87 0.64 0.92]
LR	MolDesc	[0.92 0.76 0.88]	[0.93 0.74 0.88]	[0.91 0.77 0.88]	[0.93 0.79 0.86]	[0.95 0.81 0.83]	[0.92 0.78 0.89]
	ECFP	[0.99 0.97 0.99]	[0.99 0.97 0.99]	[0.99 0.97 0.98]	[0.94 0.80 0.89]	[0.94 0.83 0.86]	[0.94 0.76 0.93]
	MACCS	[0.86 0.59 0.75]	[0.88 0.58 0.74]	[0.84 0.59 0.77]	[0.91 0.73 0.81]	[0.93 0.77 0.76]	[0.89 0.69 0.87]
	KRFP	[0.93 0.84 0.93]	[0.94 0.82 0.94]	[0.93 0.87 0.92]	[0.94 0.79 0.88]	[0.96 0.82 0.82]	[0.93 0.75 0.93]

LR: Logistic Regression, MLP: Multi-Layer perceptron, MolDesc: Molecular Descriptor, RF: Random Forest, SVM: Support Vector Machine, XGB: XGBoost

Supplementary Table 5. Affinity prediction: Weighted and class specific performance metrics after y-randomization using XGB

	Training data set Weighted [no-affinity low-affinity high-affinity]			Test data set Weighted [no-affinity low-affinity high-affinity]		
**Descriptors**	**F1 score**	**Precision**	**Recall**	**F1 score**	**Precision**	**Recall**
MolDesc	0.98 [0.99 0.98 0.98]	0.98 [1. 0.98 0.97]	0.98 [0.98 0.98 1.]	0.48 [0.69 0.41 0.32]	0.48 [0.61 0.52 0.39]	0.50 [0.80 0.34 0.27]
ECFP	0.91 [0.91 0.90 0.88]	0.92 [0.98 0.87 0.79]	0.91 [0.85 0.93 0.98]	0.51 [0.6 0.37 0.37]	0.53 [0.61 0.4 0.33]	0.50 [0.59 0.35 0.42]
MACCS	0.87 [0.88 0.85 0.85]	0.88 [0.97 0.79 0.77]	0.86 [0.800.92 0.94]	0.50 [0.5 0.43 0.33]	0.51 [0.59 0.38 0.30]	0.50 [0.43 0.5 0.37]
Klekota Roth	0.74 [0.78 0.73 0.72]	0.76 [0.89 0.69 0.61]	0.74 [0.69 0.76 0.87]	0.44 [0.56 0.35 0.36]	0.47 [0.63 0.33 0.32]	0.43 [0.5 0.38 0.42]

MolDesc: Molecular Descriptor, XGB: XGBoost

Supplementary Table 6. Potency prediction: Weighted and class specific performance metrics after y-randomization using XGB

	Training data set<br>Weighted [no-affinity low-affinity high-affinity]			Test data set<br>Weighted [no-affinity low-affinity high-affinity]		
**Descriptors**	**F1 score**	**Precision**	**Recall**	**F1 score**	**Precision**	**Recall**
MolDesc	0.98<br>[0.98 0.97 0.98]	0.98<br>[0.98 0.98 0.98]	0.98<br>[0.99 0.96 0.98]	0.48<br>[0.64 0.29 0.45]	0.48<br>[0.56 0.43 0.47]	0.50<br>[0.74 0.22 0.44]
ECFP	0.88<br>[0.89 0.84 0.88]	0.89<br>[0.83 0.96 0.90]	0.88<br>[0.97 0.74 0.85]	0.51<br>[0.62 0.22 0.43]	0.53<br>[0.52 0.38 0.47]	0.53<br>[0.75 0.15 0.39]
MACCS	0.88<br>[0.89 0.86 0.87]	0.89<br>[0.85 0.91 0.90]	0.88<br>[0.94 0.81 0.84]	0.48<br>[0.60 0.39 0.46]	0.48<br>[0.54 0.49 0.50]	0.49<br>[0.69 0.32 0.42]
Klekota Roth	0.74<br>[0.80 0.67 0.73]	0.78<br>[0.68 0.90 0.85]	0.75<br>[0.96 0.53 0.64]	0.45<br>[0.61 0.24 0.35]	0.46<br>[0.49 0.52 0.46]	0.47<br>[0.81 0.16 0.29]

MolDesc: Molecular Descriptor, XGB: XGBoost

Supplementary Table 7. Explanation of molecular descriptors

Descriptor Class	Feature	Explanation
Apol	apol	Sum of the atomic polarizabilities (including implicit hydrogens)
Atom type electrotopological state	LipoaffinityIndex	Lipoaffinity index
	maxHaaCH	Maximum atom-type H E-State: :CH:
	maxHCsats	Maximum atom-type H E-State: H bonded to B, Si, P, Ge, As, Se, Sn or Pb
	maxwHBa	Maximum E-States for weak Hydrogen Bond acceptors
	maxHBint4	Maximum E-State descriptors of strength for potential Hydrogen Bonds of path length 4
	mindssC	Minimum atom-type E-State: =C<
	SaaCH	Sum of atom-type E-State: :CH:
	SHaaCH	Sum of atom-type H E-State: :CH:
	SHBint5	Sum of E-State descriptors of strength for potential hydrogen bonds of path length 5
Autocorrelation	AATS6i	Average Broto-Moreau autocorrelation - lag 6 / weighted by first ionization potential
	AATSC1m	Average centered Broto-Moreau autocorrelation - lag 1 / weighted by mass
	AATSC3p	Average centered Broto-Moreau autocorrelation - lag 3 / weighted by polarizabilities
	AATSC4e	Average centered Broto-Moreau autocorrelation - lag 4 / weighted by Sanderson electronegativities
	ATS3s	Broto-Moreau autocorrelation - lag 3 / weighted by I-state
	ATSC0i	Centered Broto-Moreau autocorrelation - lag 0 / weighted by first ionization potential
	ATSC3e	Average centered Broto-Moreau autocorrelation - lag 3 / weighted by Sanderson electronegativities
	ATSC3p	Centered Broto-Moreau autocorrelation - lag 3 / weighted by polarizabilities
	ATSC4v	Centered Broto-Moreau autocorrelation - lag 4 / weighted by van der Waals volumes
	GATS4p	Geary autocorrelation - lag 4 / weighted by polarizabilities
	GATS8s	Geary autocorrelation - lag 8 / weighted by I-state

Descriptor Class	Feature	Explanation
	MATS1e	Moran autocorrelation - lag 1 / weighted by Sanderson electronegativities
	MATS5m	Moran autocorrelation - lag 5 / weighted by mass
Barysz matrix	SpMAD_Dzs	Spectral mean absolute deviation from Barysz matrix / weighted by I-state
	SpMAD_DzZ	Spectral mean absolute deviation from Barysz matrix / weighted by atomic number
	VE3_D	Logarithmic coefficient sum of the last eigenvector from Barysz matrix / weighted by atomic number
	VR1_Dzi	Randic-like eigenvector-based index from Barysz matrix / weighted by first ionization potential
	VR2_Dzs	Normalized Randic-like eigenvector-based index from Barysz matrix / weighted by I-state
	VR3_Dzs	Logarithmic Randic-like eigenvector-based index from Barysz matrix / weighted by I-state
BCUT	BCUTw-1l	nhigh lowest atom weighted BCUTS
Burden modified eigenvalues	SpMAX1_Bhm	Smallest absolute eigenvalue of Burden modified matrix - n 1 / weighted by relative mass
	SpMax1_Bhv	Largest absolute eigenvalue of Burden modified matrix - n 1 / weighted by relative van der Waals volumes
	SpMax4_Bhv	Largest absolute eigenvalue of Burden modified matrix - n 4 / weighted by relative van der Waals volumes
	SpMin1_Bhe	Smallest absolute eigenvalue of Burden modified matrix - n 1 / weighted by relative Sanderson electronegativities
	SpMin2_Bhm	Smallest absolute eigenvalue of Burden modified matrix - n 2 / weighted by relative mass
	SpMin3_Bhm	Smallest absolute eigenvalue of Burden modified matrix - n 3 / weighted by relative mass
	SpMin3_Bhs	Smallest absolute eigenvalue of Burden modified matrix - n 3 / weighted by relative I-state
	SpMin4_Bhm	Smallest absolute eigenvalue of Burden modified matrix - n 4 / weighted by relative mass
	SpMin5_Bhv	Smallest absolute eigenvalue of Burden modified matrix - n 5 / weighted by relative van der Waals volumes
Chi cluster	VC-5	Valence cluster, order 5
Crippen logP and MR	CrippenLogP	Crippen's LogP
Information content	IC3	Information content index (neighborhood symmetry of 3-order)
Molecular distance edge	MDEC-22	Molecular distance edge between all secondary carbons
	MDEC-33	Molecular distance edge between all tertiary carbons
Molecular linear free energy relation	MLFER_BH	Overall or summation solute hydrogen bond basicity
Topological charge	GGI7	Topological charge index of order 7
	GGI8	Topological charge index of order 8
Topological distance matrix	VR1_D	Randic-like eigenvector-based index from topological distance matrix

Supplementary Table 8. Comparison of model predictions with published experimental results

Name	Prediction (probability)	Experimental outcome	Reference
**Affinity**		**Ki**	
JWH-018	High affinity (87%)	1.22 nM	Brents et al. 2011
4-OH indole JWH-018	High affinity (58%)	2.65 nM	Brents et al. 2011
5-OH indole JWH-018	High affinity (68%)	4.22 nM	Brents et al. 2011
6-OH indole JWH-018	High affinity (66%)	17.21 nM	Brents et al. 2011
7-OH indole JWH-018	High affinity (68%)	20.79 nM	Brents et al. 2011
N-Pentanoic acid JWH-018	No affinity (67%)	>10’000 nM	Brents et al. 2011
AM-2201	High affinity (83%)	0.395 nM	Chimalakonda et al. 2012
**Potency**		**EC_{50}**	
JWH-018	High potency (74%)	42.41 nM	Åstrand et al. 2025
2-OH indole JWH-018	No potency (76%)	--	Åstrand et al. 2025
4-OH indole JWH-018	Low potency (57%)	83.58 nM	Åstrand et al. 2025
5-OH indole JWH-018	Low potency (86%)	1132.00 nM	Åstrand et al. 2025
6-OH indole JWH-018	Low potency (82%)	459.90 nM	Åstrand et al. 2025
7-OH indole JWH-018	Low potency (92%)	--	Åstrand et al. 2025
4-OH pentyl JWH-018	Low potency (87%)	288.30	Åstrand et al. 2025
5-OH indole JWH-018	Low potency (80%)	177.90	Åstrand et al. 2025
N-Pentanoic acid JWH-018	No potency (69%)	--	Åstrand et al. 2025
THJ-2201	High potency (71%)	30.65	Åstrand et al. 2025
AM-2201	High potency (78%)	36.43 nM	Åstrand et al. 2025
THJ-018	Low potency (43%)	48.8 nM	Åstrand et al. 2025

High affinity (Ki < 10 nM), low affinity (1’000 nM > Ki ≥ 100 nM), no affinity (Ki > 4000 nM)
High potency (EC50 < 50 nM), low potency (2’000 nM > EC50 ≥ 200 nM), no potency (EC50 > 6000 nM)
